# The dentate gyrus efficiently converges LEC and MEC inputs into multimodal, highly specific and reliable environmental representations

**DOI:** 10.1038/s41593-026-02240-0

**Published:** 2026-03-27

**Authors:** Thibault Cholvin, Marlene Bartos

**Affiliations:** Institute for Physiology I, University of Freiburg, Medical Faculty, Freiburg, Germany

**Keywords:** Spatial memory, Cellular neuroscience, Hippocampus

## Abstract

The entorhinal cortex–hippocampal network plays a key role in the processing, storage and retrieval of contextual information. However, how convergent multimodal information provided by the lateral and medial entorhinal cortex (LEC and MEC) is represented by granule cells (GCs), the principal cells of the dentate gyrus (DG), remains poorly understood. In this study, we employed two-photon calcium imaging of LEC and MEC projections in the DG together with GC population activity in mice navigating familiar and novel virtual environments over five consecutive days. We found that LEC inputs primarily convey olfactory information, whereas MEC inputs provide rich context-specific information to the DG. Although environmental representations rapidly emerged in LEC and MEC projections upon exposure to novel environments and remained stable over time, representations in the DG improved gradually and required repeated exposure to stabilize. Our findings suggest that the convergence of rapidly emerging, high-dimensional LEC and MEC inputs into a sparse and slowly evolving GC population code provides an energy-efficient mechanism for generating multimodal, modality-specific and context-specific representations in the DG.

## Abstract (web summary)

This study reveals how the dentate gyrus gradually learns to integrate olfactory and spatial signals from the entorhinal cortex, transforming them into sparse and highly specific representations that improve with experience.

## Main

Memories of distinct experiences form the foundation of individual life stories by linking events to their spatial and temporal contexts. Principal cell assemblies in the hippocampus play a key role in episodic memory, and their reactivation is thought to reinstate associated information^1–3^. At the population level, hippocampal principal cells form spatial maps representing features of the external world, including locations (place cells) and landmarks, supporting allothetic navigation and goal-oriented behavior^4,5^. The entorhinal cortex (EC) is the primary source of contextual information to the hippocampus^6,7^, and experience-dependent modifications of spatial representations occur in both regions in response to novelty or environmental change^8–12^. However, how EC-driven inputs relate to local hippocampal representations remains unresolved. As the canonical input gate of the trisynaptic circuit, the DG is ideally positioned to integrate EC signals and relay contextual representations to downstream hippocampal areas.

The main EC projection to the DG arises from layer II (LII) neurons in the LEC and MEC^13^. LEC LII fan and pyramidal cells receive olfactory information from the olfactory bulb and piriform cortex^14^ and transmit odor identity and intensity signals to GC dendrites^15^. Beyond olfaction, the LEC also responds to visual and auditory stimuli^16^, often associated with objects^17,18^, but with weaker spatial specificity than the MEC^19^. By contrast, the MEC is enriched in spatially modulated cell types, including grid cells^20^ and place-like cells^10^. Many LII MEC principal cells projecting to the DG are modulated by running speed, head direction and environmental boundaries^21,22^, supporting their role in allocentric mapping and path integration^17,18,23^. LEC and MEC axons terminate in distinct sublayers of the DG molecular layer^16,22,24^, putting GCs in ideal position to integrate these input streams. Although remapping has been observed in both EC subdivisions^10–12^, most studies examined representations on a single day, leaving the temporal emergence and stability of EC representations largely unexplored.

To elucidate how LEC and MEC information is transformed in the DG during learning, we performed longitudinal two-photon calcium imaging of LEC/MEC axonal inputs and GC populations in head-fixed mice navigating familiar and novel virtual environments across five consecutive days. This approach allowed us, to our knowledge for the first time, to track entorhinal input dynamics over time and directly relate them to GC population activity. We characterized how each region encodes space, objects and odors, revealing that LEC and MEC predominantly convey olfactory and spatial information, respectively. These rich entorhinal signals are progressively integrated into a sparse, high-specificity GC code. Notably, while novel environment evokes immediate context representations in both LEC and MEC, the DG gradually refines these inputs over days of experience, ultimately generating a cohesive and stable representation of the learned context. We propose that this multiday transformation reflects a circuit mechanism supporting energy-efficient formation of long-lasting memories of complex spatial and sensory experiences.

## Results

### Multiday axonal recordings of LEC and MEC projections to the DG in familiar and novel virtual environments

**Fig. 1:**
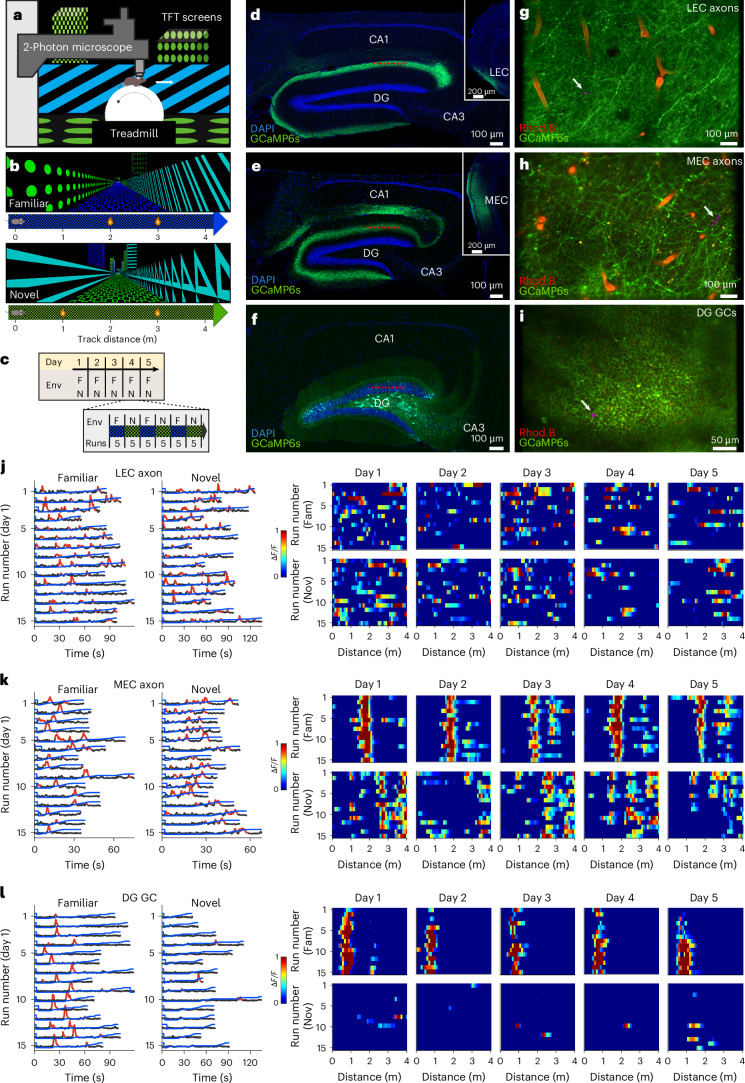
Exp1: two-photon calcium imaging of either MEC or LEC axonal projections to the hippocampus or DG GCs in mice navigating through familiar and novel virtual environments on five consecutive days. **a**, Schematic of the virtual reality setup for head-fixed mice running on a spherical treadmill. **b**, Schematic of familiar (top) and novel (bottom) 4-m-long virtual environments used in this experiment. Yellow drops indicate locations of soy milk rewards. **c**, Imaging timeline: five consecutive days of imaging in both contexts, 30 runs per day. **d**−**f**, Representative confocal images showing GCaMP6s-labeled axonal projections from the LEC (**d**) or MEC (**e**) to the DG or GCs (**f**). Blue, DAPI staining. Red dotted lines, imaging planes. **g**−**i**, Five-days average GCaMP6s fluorescence of either LEC (**g**) or MEC (**h**) axonal projections to the DG or of GCs (**i**), recorded in vivo using two-photon laser scanning microscopy. Rhodamine B was injected intraperitoneally to label blood vessels in red, facilitating identification of LEC/MEC axons during multiday imaging. White arrow points at the ROI of a single axonal projection (**g** and **h**) or a GC (**i**), of which the activity is depicted in **j**, **k** and **l**, respectively. **j**−**l**, Left panels, raw calcium traces (gray) with significant transients (red) and linear track position (blue) over time from a single LEC (**j**) or MEC (**k**) axon projection or a single GC (**l**) on day 1 of imaging. The same axons or cells were imaged over five consecutive days in both familiar and novel environments. Right panels, calcium activity over track distance of the same LEC (**j**) or MEC (**k**) axonal projection or GC (**l**) in a familiar (upper row) and a novel (bottom row) environment. Env, environment; F, familiar; Fam, familiar; N, novel; Nov, novel; Rhod.B, Rhodamine B.

**Extended Data Fig. 1:**
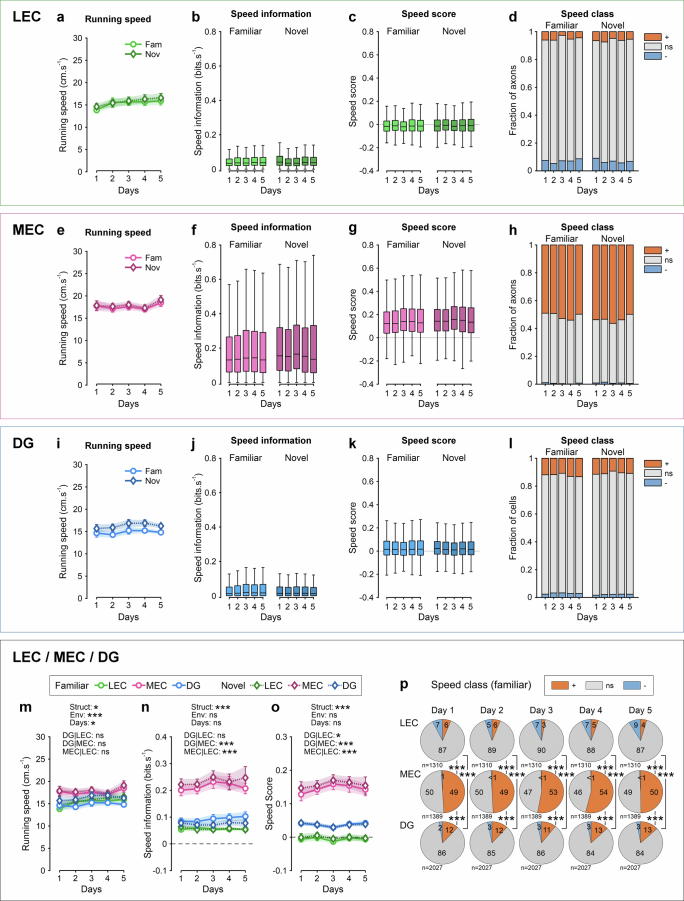
Speed modulation of LEC/MEC axons in the DG and GCs. **a, e, i** Mean running speed (excluding resting periods) of imaged mice, depending on the day (1-5) and the environment (familiar/novel). **b, f, j** Speed information of LEC- or MEC axon terminals in the DG or GCs depending on the day and environment. Speed information was calculated similarly to spatial information (see Methods) based on the average calcium activity per speed bin. **c, g, k** Speed score of LEC- or MEC axon terminals or GCs depending on the day and environment. The speed score is defined as the correlation coefficient between activity (ΔFoT, significant transients only) and running speed. **d** Fraction of LEC or MEC axon terminals or GCs of each speed class (positively, negatively or non-modulated by speed) depending on the day and environment. Axon/cell were assigned a speed class by shuffling y traces (position of the animal along the track) of the original dataset and computing the speed score of the resulting shuffled dataset. This procedure was repeated 1000 times, and the *P* value was determined as the fraction of shuffled datasets in which the speed score was higher than the speed score of the original dataset. If *P* < 0.05, the axon/cell was considered positively speed-modulated (speed class ‘+’) if its speed score was above or equal to the 99^th^ percentile of the shuffled data; if its speed score was below or equal to the 1^st^ percentile of shuffled data, it was considered negatively speed modulated (speed class ‘-‘). If none of these conditions were fulfilled, the axon/cell was considered not to be speed modulated (speed class ‘ns’). **m-o** Running speed (**m**), speed information (**n**) and speed score (**o**) of LEC/MEC/DG mice depending on the day and the environment. **p** Fraction of LEC/MEC axons and GCs positively (‘+’), negatively (‘-‘) or not significantly speed modulated (that is, speed class) over days in the familiar environment. **m-o** Two-way repeated measures ANOVAs, Tukey’s post-hoc test. **p** χ^2^ test (one-sided) for population overlap. Values indicate the number of cells. Lines with shadows indicate mean ± SEM. Boxes, 25th to 75th percentiles; bars, median; whiskers, 99% range. ns, not significant; *P < 0.05; **P < 0.01; ***P < 0.001. For **a,e,i,m-o**, LEC, *n* = 12; MEC, *n* = 15; DG, *n* = 14 (datasets); for **b-d,f-h,j-l,p**, LEC, *n* = 1310; MEC, *n* = 1389; DG, *n* = 2027 (axons/cells). For exact p values, see Supplementary Table 1.

**Extended Data Fig. 2:**
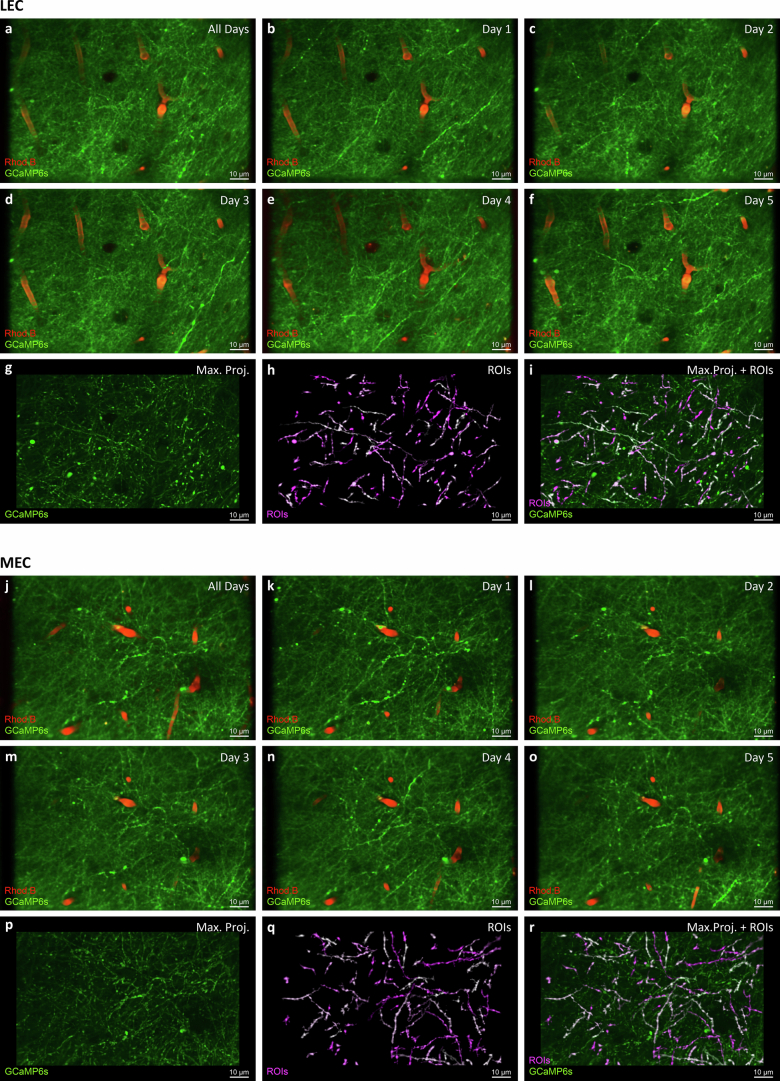
Approach applied to perform 5-days 2-Photon imaging of LEC/MEC axons projecting to the DG. **a** Mean image of the Field of View (FoV) used for all recording sessions (5 days, 150 runs) of LEC axons from experiment 1. Note the red labelling of blood vessels (Rhodamine B) used to ease the re-identification of the same FoV day after day and the positioning procedure required to find and image the exact same axon terminals over 5 consecutive days. **b-f** Mean images of the same FoV over the 5 days, day-by-day. **g** Maximal projection of the same FoV, all days combined. **h** Regions of interest (ROIs) identified in this dataset, using Suite2p and after manual curation; for more details about ROIs selection (and rejection), see Supplementary Fig. 3. **i** Selected ROIs superimposed on maximal projection image. **j-r** Similar to **a-i** for a dataset of MEC axonal projections. For details, see Methods.

To assess the emergence and stability of information conveyed by LEC and MEC projections to the DG, we performed two-photon calcium imaging of these inputs in mice navigating 4-m-long virtual environments over five consecutive days (Fig. 1a,b). Imaging sessions started after approximately 10 days of familiarization to one environment. Each day, mice alternatively ran through familiar and novel environments in blocks of five runs (Fig. 1c and Supplementary Fig. 1), similarly well in both contexts (Extended Data Fig. 1a,e,i,m), and licked preferentially in reward zones (Supplementary Fig. 2). To record EC inputs, mice were injected in either LEC or MEC with adeno-associated viruses (AAVs) inducing axon-enriched expression of GCAMP6s. As expected, labeled axons were observed in the outer molecular layer in LEC-injected mice (Fig. 1d) and in the middle molecular layer in MEC-injected mice (Fig. 1e). To reliably identify the same fields of view across days, we intraperitoneally injected Rhodamine B before each recording session to label blood vessels (Fig. 1g,h,i and Extended Data Fig. 2), and regions of interest (ROIs) detected by Suite2p were manually curated to ensure stable tracking (Fig. 1j,k and Supplementary Fig. 3). In a separate cohort, GC activity was recorded after AAV1-GCaMP6s injection into the dorsal DG (Fig. 1f,i,l). Peak amplitude and baseline noise remained stable across days in all groups (Supplementary Fig. 4). This experiment comprised 41 datasets (12 LEC, 15 MEC and 14 GC datasets), totaling 1,310, 1,389 and 2,027 axons/cells, respectively.

### Activity and spatial tuning of LEC and MEC inputs in the DG improve less with learning than their target GCs

**Fig. 2:**
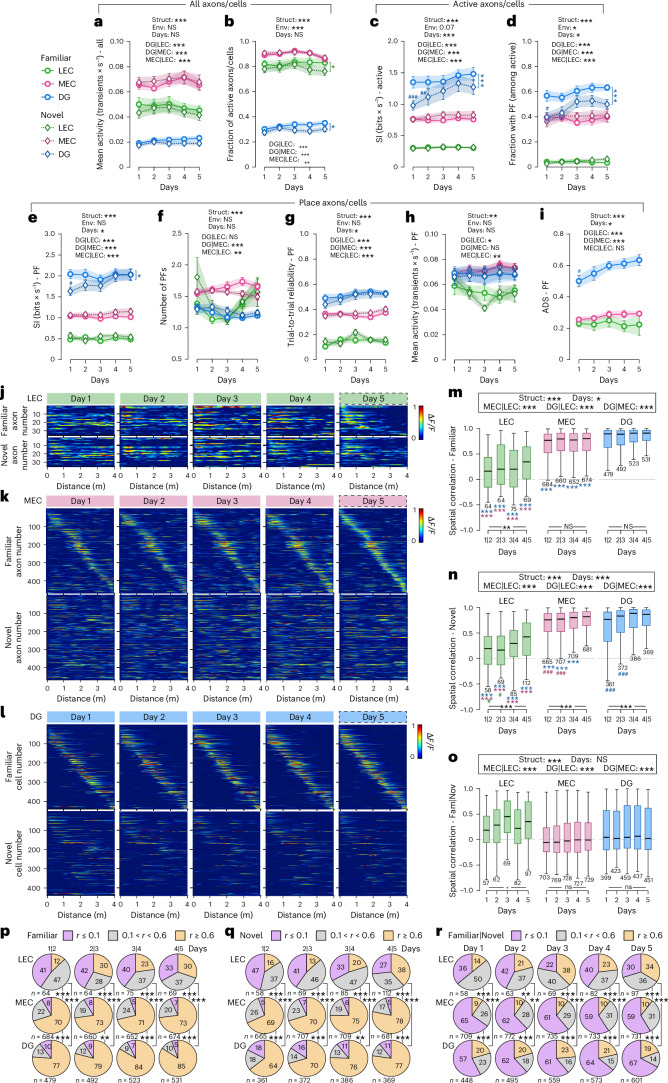
LEC and MEC axonal projections to the DG and GC populations show substantially distinct activity patterns and spatial tuning characteristics. **a**,**b**, Mean activity (transients × s^−1^, **a**) and fraction of active (>1 transients per minute, **b**) axons/cells of all LEC (green) and MEC (pink) axonal projections in the DG as well as GCs (blue) recorded over five consecutive days in both familiar (light colors, circle marker) and novel (dark colors, diamond marker) environments. **c**, **d**, SI (bits × s^−1^) of tuned LEC/MEC axons as well as GCs (**c**) and fraction of axons/cells with one or multiple PFs (**d**). **e**−**i**, SI (bits × s^−1^, **e**), number of PFs (**f**), trial-to-trial reliability (**g**), mean activity (transients × s^−1^, **h**) and ADS (**i**) of LEC/MEC axons as well as DG GCs with PFs. **j**−**l**, Activity maps of axons with a PF projecting from the LEC (**j**) or the MEC (**k**) to the DG or of place GCs (**l**) imaged over five consecutive days in a familiar and a novel environment. Sorting was according to the axon/cell activity on day 5 in the familiar environment. **m**−**o**, Spatial correlation of axons/cells with a PF on two consecutive days in familiar (**m**) and novel (**n**) environments or on individual days between the familiar and novel environment (**o**). **p**−**r**, Fraction of place axons/cells (same ones as in **m**−**o**, respectively) showing high (*r* ≥ 0.6, orange), medium (0.1 < *r* < 0.6, gray) or low (*r* ≤ 0.1, purple) PF correlations between two consecutive days in the familiar (**p**) or novel (**q**) environment and between familiar and novel environments for each day (**r**). For complementary analyses, including comparisons between environments within each structure, see Extended Data Fig. 3. **a**−**i**, Three-way (**a**−**h**) or two-way (**i**) repeated-measures ANOVAs, Tukey’s post hoc test. **m**−**o**, Two-way ANOVAs after alignment and ranking (Methods), Tukey’s post hoc test. **p**−**r**, *χ*^2^ test for population overlap. Forty-one datasets from 23 mice in total; LEC, 12 datasets; MEC, 15 datasets; DG GCs, 14 datasets. Lines with shadows indicate mean ± s.e.m. Boxes, 25th to 75th percentiles; bars, median; whiskers, 99% range. Values indicate the number of cells. NS, not significant; ^#^/*P < 0.05; ^##^/**P < 0.01; ^###^/***P < 0.001. Black stars indicate main effects; colored stars, differences between environments within a group (**a**−**i**) or between two groups for the same day comparison (**m**−**o**); colored hashtags, differences between days 1, 2, 3 or 4 and day 5 within the same group and environment (**a**−**i**) or between ‘1|2’, ‘2|3’ or ‘3|4’ and ‘4|5’ (**m**−**n**). For exact *P* values, see Supplementary Table 1. Env, environment; Fam, familiar; Nov, novel; Struct, structure.

We first quantified mean activity and spatial information (SI) of LEC and MEC inputs and compared them with GCs. Mean activity and the fraction of active units were highest in MEC inputs, intermediate in LEC inputs and lowest in GCs in both contexts (Fig. 2a,b). Commensurate with the idea that the LEC makes a minimal contribution to spatial representations^6,19^, LEC axons exhibited the lowest SI, followed by MEC inputs, whereas GCs showed the highest SI (Fig. 2c). Notably, SI increased progressively across days in GCs exposed to a novel environment but peaked on day 1 and remained stable in LEC and MEC inputs (Fig. 2c,e). The fraction of units with place fields (PFs) was negligible for LEC inputs, markedly larger for MEC fibers and highest for GCs (Fig. 2d), with the fraction of GCs with PFs increasing over days in the novel context (Fig. 2c,d). MEC inputs more frequently exhibited multiple PFs than LEC inputs or GCs (Fig. 2f). To examine the stability of spatial representations, we computed the trial-to-trial reliability, which was lowest for LEC inputs, intermediate for MEC axons and highest for GCs (Fig. 2g) and improved over days only in GCs. Thus, spatially tuned EC inputs emerge rapidly and remain stable, whereas DG representations gradually improve in SI and reliability with experience.

### The fraction of LEC/MEC inputs in the DG and GCs with consistent PFs increases over days during novel context learning

**Extended Data Fig. 3:**
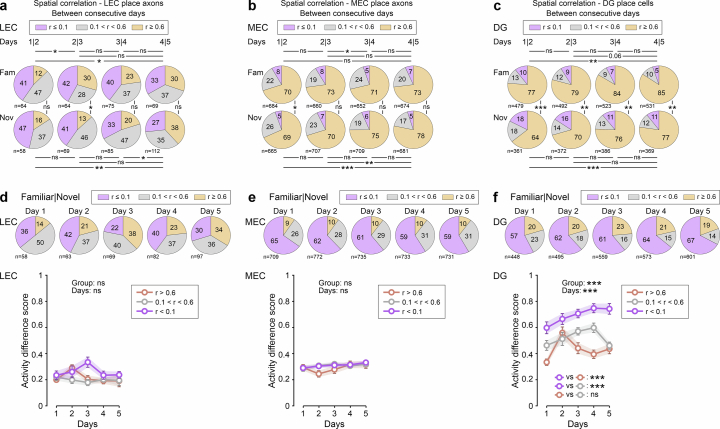
Spatial correlations categories of axons/cells with PFs and activity difference scores depending on the spatial correlation between environments. **a-c** Fraction of axons with a place field (same as in Fig. 2p,q) for LEC axons (**a**), MEC axons (**b**) and GCs (**c**) showing high (*r* ≥ 0.6, orange), medium (0.1 < *r* < 0.6, grey) or low (*r* ≤ 0.1, purple) place field correlations between two consecutive days in familiar (upper row) or novel (bottom row) environments. **d-e** Top panels, same data as in Fig. 2r (fraction of place axons/cells showing high (r ≥ 0.6, orange), medium (0.1 < r < 0.6, grey) or low (r ≤ 0.1, purple) place field correlations between familiar and novel environments for each day). Bottom panels, activity difference score depending on the three sub-categories of axons/cells identified above. **a-c** χ2 test (one-sided) for population overlap. **d-f** Two-way repeated measures ANOVAs, Tukey’s post hoc test. Lines with shadows indicate mean ± SEM. ns, not significant; ***P < 0.001. For exact p values, see Supplementary Table 1.

To examine PF consistency, we computed day-to-day PF correlations (Fig. 2j−l). PF correlations remained low for LEC inputs in both contexts (Fig. 2m,n). MEC axons showed higher PF consistency than LEC inputs but lower than GCs, and PF consistency increased over days in the novel environment for MEC inputs and GCs but not in the familiar context (Fig. 2m,n). Categorizing units by low, intermediate and high PF correlations revealed a larger fraction of highly consistent units in GCs than in MEC/LEC inputs in both contexts (Fig. 2p,q and Extended Data Fig. 3a−c). This fraction increased selectively in GCs in the novel environment (Fig. 2q).

### LEC/MEC inputs and GCs show different preferences for rate versus global remapping

To quantify context discrimination, we computed the activity difference score (ADS). Although mean activity of MEC inputs and GCs with PFs was similar (Fig. 2h), ADS was substantially higher in GCs and increased across days, whereas it remained steadily low in both MEC and LEC inputs (Fig. 2i). PF cross-correlations between environments revealed strong global remapping in MEC inputs and GCs, with median correlations near zero (Fig. 2o,r). By contrast, the small fraction of spatially tuned LEC inputs showed higher PF correlations, indicating weak context specificity. Approximately 60% of MEC inputs and GCs exhibited strong global remapping (*r* ≤ 0.1), whereas approximately 20% of GCs showed high PF correlations, indicating context generalization (*r* ≥ 0.6; Fig. 2r). ADS analysis across remapping categories (Extended Data Fig. 3d−f) showed no significant difference between LEC and MEC axons, whereas GCs with the least spatial overlap showed the highest ADS. This elevated ADS can be explained by the sparser representation in GCs relative to EC inputs. Moreover, within the high-correlation category, ADS was markedly higher in GCs than in LEC/MEC inputs, consistent with stronger rate-remapping-based discrimination in GCs. Together, these results indicate combined rate and global remapping in GCs, predominant global remapping in MEC inputs and limited discrimination by spatially tuned LEC axons.

### Decoding of spatial location and context improves over days in GCs but not in EC inputs

**Fig. 3:**
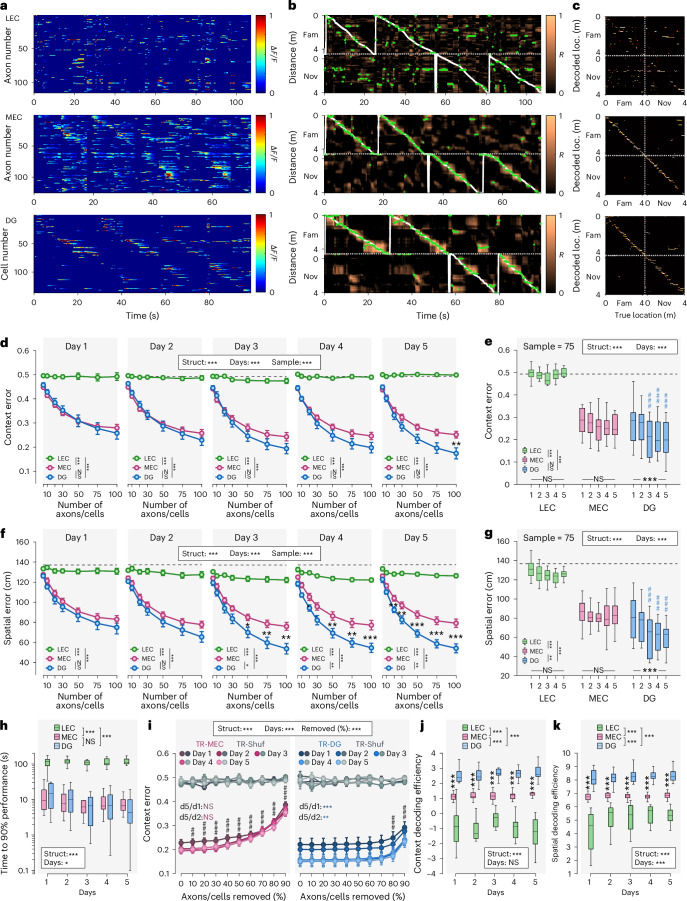
Decoding of space and context from neuronal activity confirms steady spatial properties of MEC inputs while DG GCs’ contextual representation progressively improves over days, leading to more precise yet highly efficient spatial decoding. **a**−**c**, Decoding examples: top, LEC; middle, MEC; bottom, DG GCs. **a**, Activity of LEC and MEC axon and GC ensembles is plotted over time in four subsequent laps (the first two in the familiar context and the last two in the novel context). **b**, Decoder output. White line denotes true position of the mouse and the green dots the predicted position. **c**, Confusion matrix of actual mouse location (*x\* axis) and maximum likelihood decoded locations (*y* axis). Upper left and lower right dotted boxes outline real location in familiar or novel environments, respectively. **d**,**e**, Contextual decoding error as a function of the number of LEC/MEC axons/GCs used simultaneously for decoding. **d**, Decoding results depend on sample size (from 5 to 100 axons/cells). **e**, Decoding errors from a single sample size of 75 LEC/MEC axons or GCs compared over days within each structure. **f**,**g**, Same as **d** and **e**, respectively, but for spatial decoding error. **h**, Average time to 90% context decoding accuracy for a fixed ensemble size of 75 axons/cells, respectively. **i**, Contextual decoding errors after removal of the least active (using transient rate) axons/cells in steps of 10% for MEC axons (left) and GCs (right). For LEC axons as well as spatial decoding, see Extended Data Fig. 4. **j**,**k**, CDE (**j**) and SDE (**k**) defined as the log of the contextual or spatial decoding accuracy (actual decoding − shuffled (chance level) decoding) divided by the activity (transient rate), respectively. Sampling size, 75 axons/cells. **d**,**f**,**i**, Three-way repeated-measures ANOVAs; **e**,**g**,**h**,**j**,**k**, two-way repeated-measures ANOVAs; Tukey’s post hoc test. Lines with error bars indicate mean ± s.e.m. Boxes, 25th to 75th percentiles; bars, median; whiskers, 99% range. NS, not significant; ^#^/*P < 0.05; ^##^/**P < 0.01; ^###^/***P < 0.001. Black stars indicate main effects (all plots) as well as comparisons between structures (**d**−**g**,**j**,**k**) and days effect within a structure (**e**,**g**); colored stars, differences between days (**i**); hashtags, differences between day 1 and any other day (**e**,**g**) or for a specific percentage of axons/cells removed compared to baseline—that is, 0% removed (**i**). Gray dashed line shows chance level (shuffled data). For exact *P* values, see Supplementary Table 1. d, day; Fam, familiar; loc., location; Nov, novel; Shuf, shuffled; Struct, structure; TR, transient rate.

**Extended Data Fig. 4:**
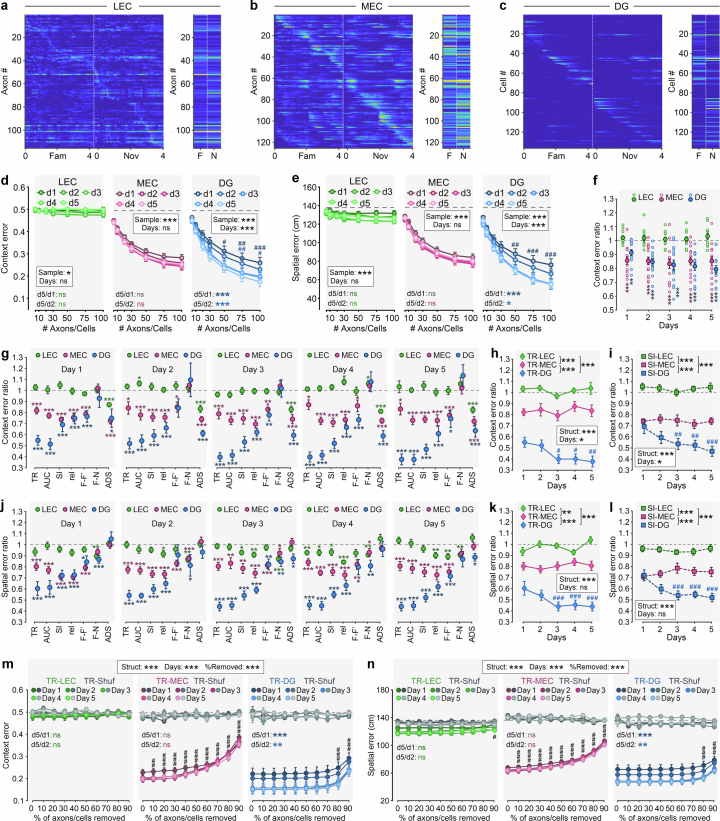
Decoding space and context from neuronal activity, Exp 1. **a-c** Illustrative examples of the different templates used for the decoding approach, constructed either from the environment-specific spatial stratified templates of activity patterns (aSC, left panel) or by using only the mean activity-rates for individual cells for each environment (aC, right panel). Templates were obtained from 115 LEC axon terminals (**a**), 125 MEC axon terminals (**b**) and 136 GCs (**c**), respectively. **d, e** Contextual (**d**) and spatial (**e**) decoding errors as a function of the number of LEC/MEC axons or GCs used simultaneously for decoding. Based on the same data as Fig. 3**d** and **f**, respectively, but focusing on the comparison between days within each structure (LEC/MEC axons or GCs). **f** Ratio of context decoding errors obtained using a template based on context-specific spatial maps (aSC) divided by context decoding errors obtained using mean activity-rate per context (aC) only (Err_aSC_/Err_aC_). **g, j** Ratio of contextual (**g**) and spatial (**j**) decoding errors from the half of the datasets with the highest values of each parameter divided by the half with lowest values, such that small values indicate high predictive value. The contextual decoding error is plotted depending on the activity (transient rate, TR), AUC rate (AUC), spatial information (SI), trial-to-trial reliability (rel), consistency among sessions in the familiar environment (F-F’), activity-map correlation between familiar and novel environments (F-N) and the activity difference score between environments (ADS). **h, i** Ratio of contextual decoding errors from the half of the datasets with the highest activity (TR, **h**) or spatial information (SI, **i**), respectively, divided by the lowest half. Same data as in **g**, but here data are plotted for a single parameter and as a function of time. **k, l** Similar to **h** and **i**, respectively, but for spatial decoding. Based on **j** data. **m, n** Ratio of decoding errors after step-wise removal of axons/cells from the pool used for decoding of context (**m**) and space (**n**) based on their activity (transient rate, TR). **d, e, h, i, k, l** Two-way repeated measures ANOVAs; **m, n** Three-way repeated measures ANOVAs; Tukey’s post-hoc test; **f, h, i, k, l** Signed Rank Test. Lines or circular data points with error bars indicate mean ± SEM. ns, not significant; #/*P < 0.05; ##/**P < 0.01; ###/***P < 0.001. Black stars indicate main effects (all plots); colored stars, differences between days (**d, e, m, n**) or comparisons to chance level (**f, g, j**); hashtags, differences between day 1 (light color) or 2 (dark color) and day 5 for a given sample size (**d, e**); differences between day 1 and any other day (**h, i, k, l**) or for a specific percentage of axons/cells removed compared to baseline, that is 0% removed (**m, n**). Grey dashed line shows chance level (shuffled data). For **d-n**, LEC, *n* = 12; MEC, *n* = 15; DG, *n* = 14 (datasets). For exact p values, see Supplementary Table 1.

We next trained a population-vector-based decoder to predict context and location^12,25,26^ (Fig. 3a−c). Using random samples of axons/cells with matched sizes, increasing ensemble size reduced decoding error for all populations (Fig. 3d,f). On day 1 in the novel environment, decoding accuracy was similar for MEC inputs and GCs but improved selectively in GCs, and this improvement was more pronounced for locations than contexts (Fig. 3d versus Fig. 3f). To examine potential amelioration over time, we fixed the sample size to 75 axons/cells, confirming that prediction errors only improved over days in GCs (Fig. 3e,g) and suggesting progressive orthogonalization of contextual representations specifically in GCs. LEC inputs provided poor decoding performance throughout (Fig. 3e,g). Comparing decoders based on spatially structured versus average activity templates revealed improved performance for MEC inputs and GCs but not for LEC inputs (Extended Data Fig. 4a−c,f), indicating that spatial remapping conveys information beyond rate changes between contexts.

Parameter-specific analyses showed that decoding in MEC inputs and GCs depended on activity level, SI, reliability and ADS, whereas ADS was the only consistent contributor for LEC inputs (Extended Data Fig. 4g−l). To examine how experience impacts these results, we focused on two parameters showing particularly high influence on DG and MEC data: the transient rate and SI (Extended Data Fig. 4h,i,k,l). Although the error ratios for context and space decoding for LEC/MEC input activity remained stable across days, they gradually improved in GCs over the first 3 days.

We further quantified the time required to reach 90% decoding performance for ensembles of 75 axons/GCs: more than 100 seconds for LEC inputs (approximately 112 seconds) but only 7.3 seconds for MEC axons and 8.5 seconds for GCs. This duration decreased across days only in GCs, from 14.9 seconds (day 1) to 4.3 seconds (day 5) (Fig. 3h). Thus, relatively small ensembles of MEC inputs and GCs are sufficient to rapidly read-out the environment, but improvement over days is specific to GCs. Removing progressively larger fractions of units revealed that GC decoding remained robust until approximately 80% of cells were removed, whereas MEC decoding declined monotonically (Fig. 3i and Extended Data Fig. 4m,n). Furthermore, context and spatial decoding errors significantly decreased over time only in GCs, reaching a stable low level on day 3 (Extended Data Fig. 4n). Finally, given that highly active LEC/MEC inputs transmit information to a GC population discharging only few action potentials during spatial navigation^27^, we asked how activity levels relate to decoding performance. We computed indices of contextual decoding efficiency or spatial decoding efficiency (CDE/SDE). Both indices were significantly higher for GCs than for LEC/MEC axons (Fig. 3j,k), indicating higher energy efficiency of GCs in conveying SI as compared to EC inputs.

### Running speed information conveyed by MEC inputs is sparsely represented in GCs

Running speed is encoded by the MEC^28,29^. Less information is available on speed coding in LEC and DG. We computed the speed information (bits × s^−1^) and the speed score (correlation coefficient between neuronal activity and running speed) of LEC/MEC inputs and GCs. Both metrics were higher for MEC as compared to LEC inputs or GCs (Extended Data Fig. 1f,g,j,k,n,o). Speed scores were also markedly higher in GCs than in LEC inputs (Extended Data Fig. 1c,g,k,o). These results were similar for both environments (Extended Data Fig. 1n,o). Classifying axons/cells into negatively, positively or non-significantly speed modulated revealed that, on average, 51% of MEC inputs, 12% of GCs and 5% of LEC inputs were positively speed modulated, whereas only 1% of MEC inputs, 3% of GCs and 7% of LEC inputs showed negative speed modulation (Extended Data Fig. 1d,h,l,p). Thus, most of the speed information reaching the DG is provided by LII MEC inputs and is unaffected by novelty.

### LEC olfactory and MEC cue information converges in the DG

**Fig. 4:**
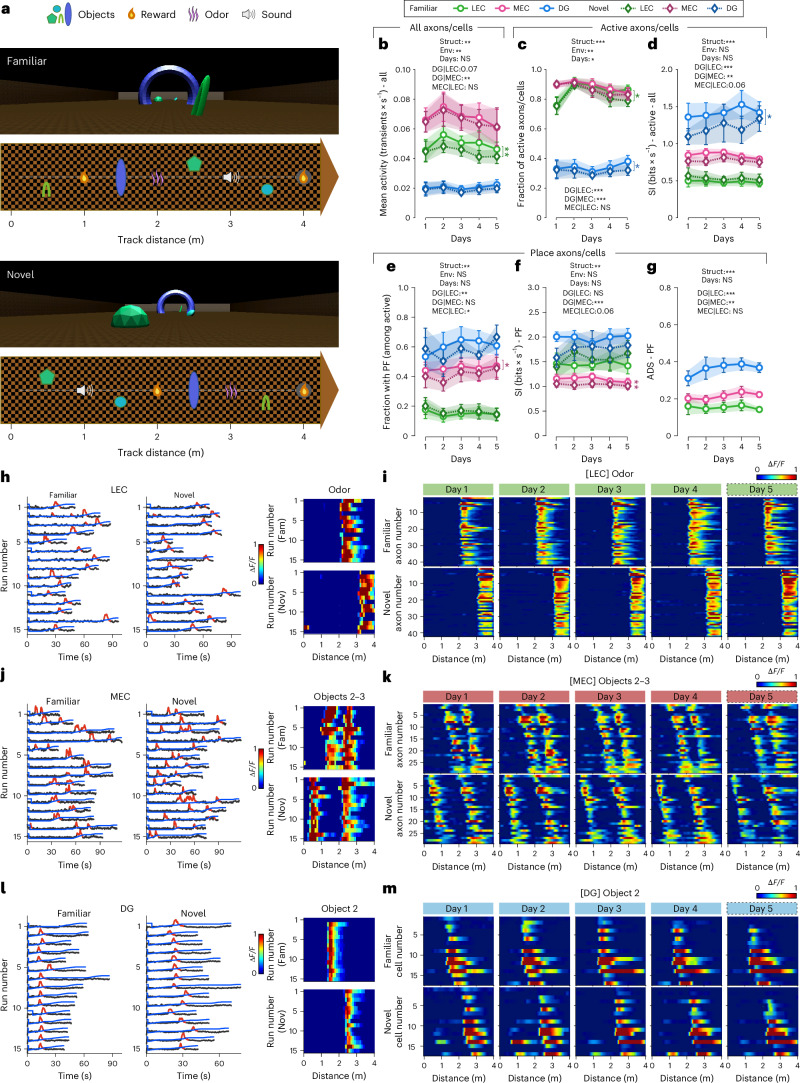
Exp2: two-photon calcium imaging of MEC/LEC axonal projections to the DG or GCs over 5 days in mice navigating through two versions of an open-field-like linear virtual environment comprising objects, rewards and odor and sound stimuli. **a**, Schematic of familiar (top) and novel (bottom) 4-m-long open-field-like linear virtual environments used in this experiment. The same box-like virtual background is used in both environments, but the sound, the odor, the first reward and four different objects are distributed in different orders (familiar/novel). **b**,**c**, Mean activity (transients × s^−1^, **b**) and fraction of active (>1 transients per minute) axons/cells (**c**) of all LEC (green) and MEC (pink) axonal projections to the DG as well as GCs (blue) recorded over five consecutive days in both familiar (light colors, circle marker) and novel (dark colors, diamond marker) environments. **d**,**e**, SI (bits × s^−1^) of active axons/cells as well as GCs (**d**) and fraction of them with one or multiple PF(s) (**e**). **f**,**g**, SI (bits × s^−1^, **f**) and ADS (**g**) of LEC/MEC axons and GCs having PF(s). **h**, Left panels, raw calcium traces (gray) with significant calcium transients (red) and linear track position (blue) over time of a single LEC cell coding for the odor on day 3. Right panels, calcium activity over track distance of the same LEC axon terminal in familiar (top) and novel (bottom) environments. **i**, Activity maps of all odor-encoding axons projecting from the LEC to the DG, imaged over five consecutive days in familiar and novel environments. Sorting according to the cellsʼ activity on day 5 in the familiar environment (five datasets from five mice). **j**,**k**, Similar to **h** and **i**, respectively, for a single MEC axon terminal projecting to the DG and encoding objects 2 and 3 (**j**) as well as the activity maps of all MEC axons showing the same pattern (**k**). **l**,**m**, Similar to **h** and **i**, respectively, for DG GC(s) encoding object 2. **b**−**g**, Three-way (**b**−**f**) or two-way (**g**) repeated-measures ANOVAs, Tukey’s post hoc test. Lines with shadows indicate mean ± s.e.m. NS, not significant; *P < 0.05; **P < 0.01; ***P < 0.001. Black stars indicate main effects; colored stars, differences between environments within a group. See also Extended Data Fig. 5 and Supplementary Fig. 5. For exact *P* values, see Supplementary Table 1. Env, environment; Fam, familiar; Nov, novel; Struct, structure.

**Extended Data Fig. 5:**
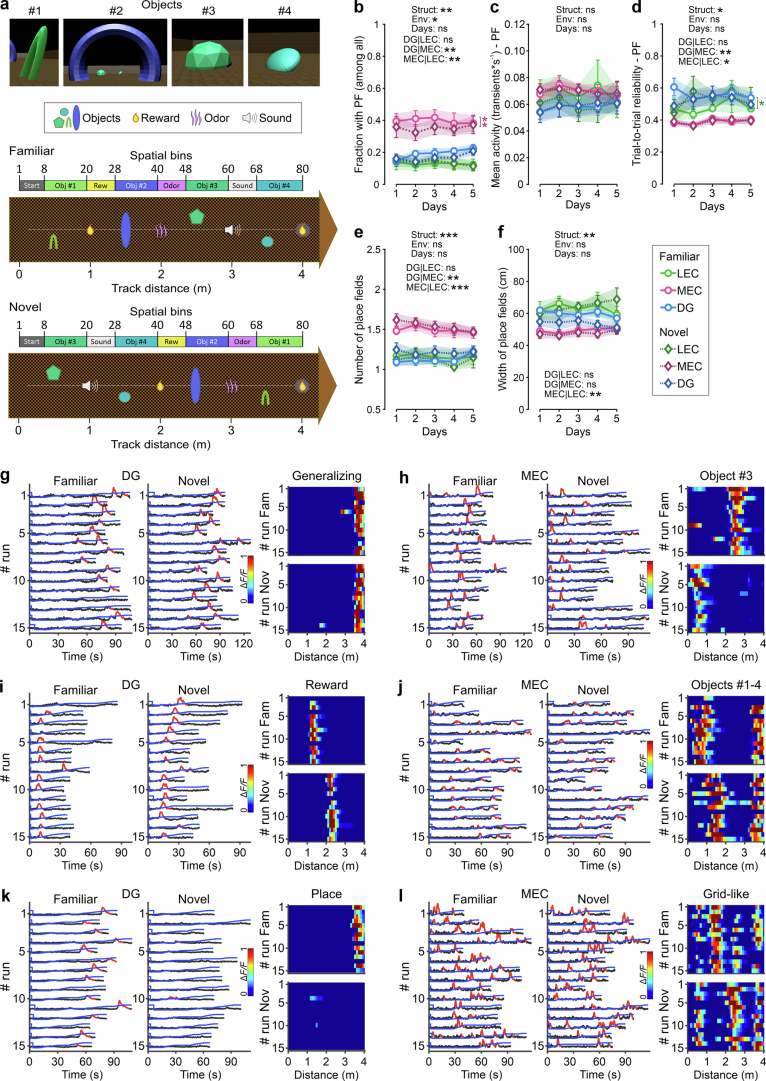
Activity properties of LEC/MEC axons and GCs and the identification of types of axons/cells coding for objects or sensory stimuli. **a** Upper panel, images of the 4 objects used in this experiment (in the familiar version of the task). Lower panel, schematic representation of the two versions of the familiar (top) and novel (bottom) 4m-long open field-like linear virtual environments, depicting the sections (spatial bins) with each of the 4 objects (light green, dark blue, dark green and turquoise) the rewards, odor and sound. The same number of bins for each section (12 for the objects, 8 for rewards, odor and sound) were used in both versions of the environment but at different orders. **b** Fraction with place field(s) among all axons/cells. **c-f** Mean activity (transients*s^−1^, **c**), mean place field trial-to-trial reliability (pairwise cross-correlations between all runs in the same environment, **d**), number of place fields (**e**) and mean place field width (**f**) of LEC/MEC axons and GCs with place field(s). **g** Left panel, raw calcium traces (grey) with significant transients (red) and linear-track positions (blue) over time of a single GC having a place field in the same location in both versions of the task, that is generalizing between the two environments. Right panels, calcium activity over track distance of the same cell in the familiar (top) and the novel (bottom) versions of the environment. **i, k** Similar to **g** for two GCs coding for the first reward site (**i**) or having a place field at the end of the familiar environment, but none in the novel one, and thus being classified as a place cell (**k**). **h, j, l** Similar to **g** for three MEC axon terminals of which the activity is specifically associated with object #3 (**h**), objects #1 and #4 (**j**) or shows grid-like activity patterns (**l**). **b-f** Three-way repeated measures ANOVAs, Tukey’s post-hoc test. Lines with shadows indicate mean ± SEM; ns, not significant; *P < 0.05; **P < 0.01; ***P < 0.001. Black stars indicate main effects; colored stars, differences between environments within a group. For **b-f**, LEC, *n* = 5; MEC, *n* = 7; DG, *n* = 5 (datasets). For exact p values, see Supplementary Table 1.

To dissociate the contribution of local sensory cues from global contextual structure, we designed a second experiment using two dissimilar versions of a linear virtual environment resembling an open field. Made of identical wall and floor patterns, they contained the same four objects (1−4) and sensory stimuli (odor, sound and reward) but differently distributed along the path (Fig. 4a and Extended Data Fig. 5a). This experimental design allowed us to ask which features of the environment (local sensory elements, their spatial configuration or global structure) are preserved or transformed in EC inputs and DG representations when sensory features are redistributed within a fixed context. Analogous to experiment 1 (Exp1; Fig. 1c), animals were initially trained on one version of the environment (familiar) and then exposed to the novel one from the start of imaging onwards, and we imaged LEC/MEC axons or GCs over 5 days (431 LEC axons, 632 MEC axons and 650 GCs in five, seven and five mice, respectively). As in Exp1, we observed the highest mean transient rate in MEC inputs, followed by LEC inputs and sparse activity in GC populations (Fig. 4b,c). SI of active axons/cells (Fig. 4d), fraction with PF (Fig. 4e) and ADS (Fig. 4g) remained consistent too, being the highest for GCs, followed by MEC and LEC inputs. Thus, the intrinsic coding properties of EC inputs and GCs appeared unchanged between open-field-like linear virtual environments (experiment 2 (Exp2)) and corridor-like linear tracks (Exp1).

**Fig. 5:**
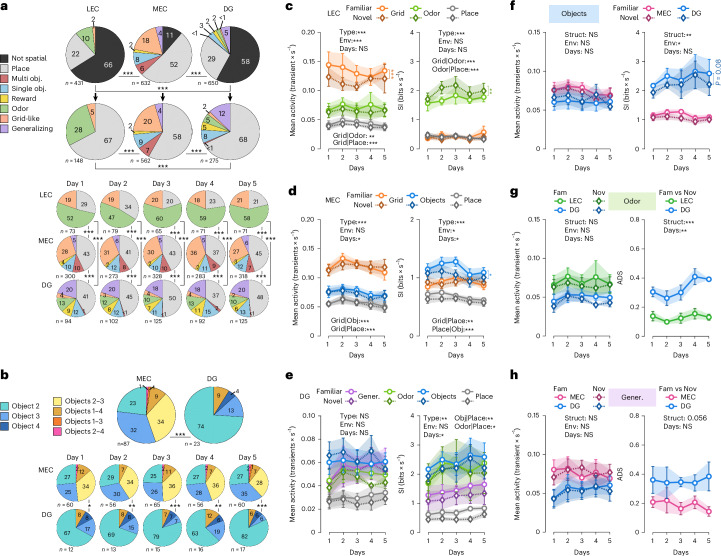
Information from LEC axon terminals (primarily encoding the odor) and MEC projections (providing spatially tuned, grid-like and object-related information to the DG) converge onto GC populations. **a**, Fraction of LEC/MEC axons and GCs generalizing between the two environments, showing grid-like activity patterns or odor-associated, reward-associated or object(s)-associated activity, having PF(s) or no spatial tuning. Upper panel, top row, neuronal populations including not spatially tuned axons/cells (black); lower row, excluding not spatially tuned axons/cells; bottom panel (three rows), fraction of axons/cells of each neuronal class identified on each of the 5 days in the familiar environment (for novel environment, see Extended Data Fig. 6). Note: non-spatial (black) refers to axons/cells that never showed a PF on any day and context. **b**, Top row, fraction of MEC axons or GCs with activity patterns associated with one or multiple objects. Note that we did not find any object-anchored axonal projection originating from the LEC. Bottom row, fraction of cells of each object type identified each day in the familiar environment (for novel environment, see Extended Data Fig. 6). **c**−**e**, Mean activity (transients × s^−1^, left panel) and SI (bits × s^−1^, right panel) of axons/cells depending on their classification identified for the imaged LEC axons (**c**), MEC projections (**d**) and GCs (**e**) (see also Extended Data Fig. 7 for trial-to-trial reliability and ADS measures). **f**−**h**, Comparisons between structures (MEC/LEC axons and GCs) of the mean activity (transients × s^−1^, left panel, **f**−**h**) and SI (bits × s^−1^, right panel, **f**) or ADS (right panel, **g**−**h**) of axons/cells encoding for objects (**f**), the odor (**h**) or those that generalized between contexts (**h**) (see Extended Data Fig. 7 for complementary measures for axon/cell populations with grid-like and place-related activity). **a**,**b**, *χ*^2^ test for population overlap. **c**−**h**, Three-way (mean activity/SI) or two-way (ADS) repeated-measures ANOVAs, Tukey’s post hoc test. Lines with shadows indicate mean ± s.e.m. NS, not significant; *P < 0.05; **P < 0.01; ***P < 0.001. Black stars indicate main effects; colored stars, differences between environments within a group. For exact *P* values, see Supplementary Table 1. Env, environment; Fam, familiar; Gener., generalizing; obj., object; Nov, novel; Struct, structure.

**Extended Data Fig. 6:**
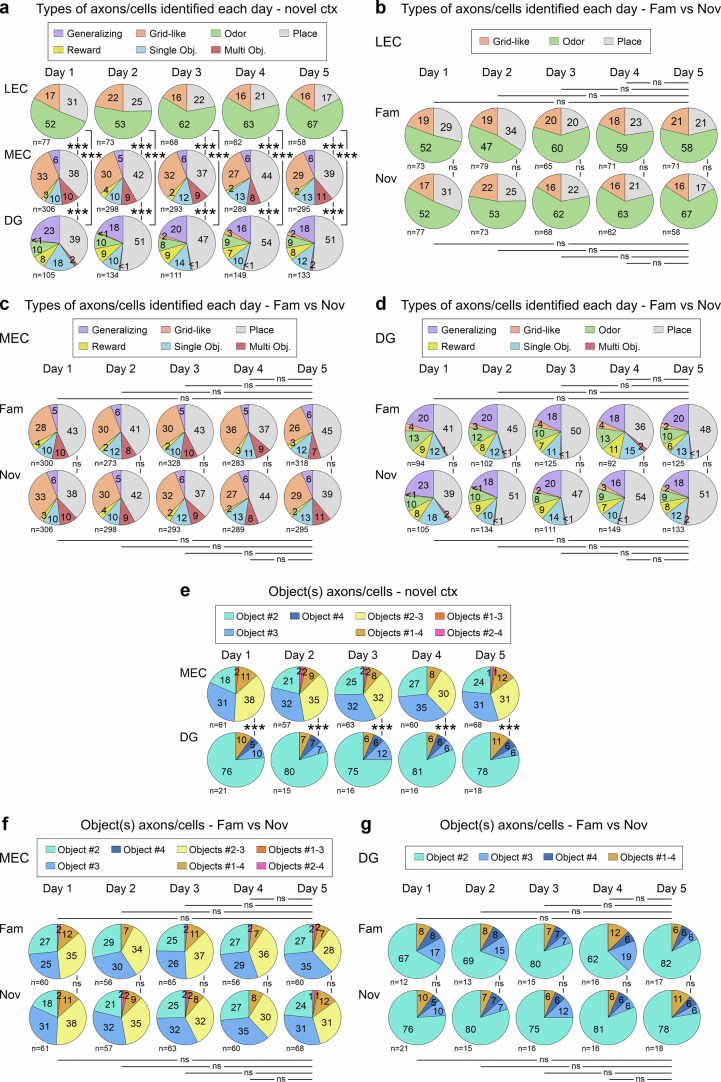
Statistical comparisons between the different classes of LEC/MEC axons and GCs encoding for objects, rewards, the odor or tone in Exp 2. **a** Fraction of axons/cells of each class identified each day in the novel environment (related to Fig. 5a, bottom panel). **b-d** Fraction of axons/cells of each class identified in the familiar (upper row) and the novel (lower row) environments on subsequent days of imaging. Comparisons of fractions between days (within familiar and novel) as well as between the two environments, for LEC (**b**) or MEC (**c**) axon terminals and GCs (**d**). **e** Fraction of MEC axons and GCs encoding each object type on each day of imaging in the novel environment (complementary to Fig. 5b (familiar environment)). **f, g** Fraction of MEC axons and GCs encoding each object type on subsequent day of imaging in the familiar (upper row) and the novel (lower row) environments. Comparisons between days (within the familiar and the novel) as well as between the two environments, for MEC (**f**) axon terminals and GCs (**g**). **a-g** χ^2^ test (one-sided) for population overlap; ns, not significant; *P < 0.05; **P < 0.01; ***P < 0.001. For exact p values, see Supplementary Table 1.

To differentiate between units representing odor (Fig. 4h,I and Supplementary Fig. 5b), reward (Extended Data Fig. 5i), sound, single objects (Fig. 4l,m, Extended Data Fig. 5h and Supplementary Fig. 5d,e), multiple objects (Fig. 4j,k and Extended Data Fig. 5j), non-periodic PFs (Extended Data Fig. 5k and Supplementary Fig. 5c) and periodic grid-like fields (Fig. 5a, Extended Data Fig. 5l and Supplementary Fig. 5f), we designed a classifier (Methods). Pooling data from all days, we found that cues and space were encoded by the activity of 34% of LEC inputs and of 42% of GCs, as opposed to 89% of MEC axons (Fig. 5a). Among the LEC axons tuned to one of these features, a marked fraction represented the odor (28%), whereas the remaining ones showed place or grid-like activity patterns (67% and 5%, respectively). In contrast to recent observations demonstrating clear representations of cues and rewards by LEC inputs to CA1 (Bowler et al.^11^), none of the LEC axons in the DG showed tuning to rewards, sound or objects or generalized between contexts. Conversely, none of MEC axons encoded for the odor, whereas substantial fractions discharged in association with one (9%) or more than one (7%) two-dimensional object and a low fraction for reward sites (2%). Most MEC inputs were spatially tuned and showed either grid or place cell characteristics (20% and 58%, respectively), with a small population generalizing between environments (4%). Notably, GCs showed high granularity in object representation with a preference to encode individual objects (8%). Reward sites and odor were also represented by some GCs (5% in both cases), and just 2% showed grid-like activity patterns, whereas most spatially tuned GCs were identified as place cells (68%). Consistent with previous reports, a substantial fraction of place GCs (12%) generalized between contexts^8,26^, markedly more than MEC inputs. None of the EC axons or GCs responded to auditory stimuli. The observed distribution of cue-tuned and spatially tuned units did not significantly change over time and was similar between the two versions of the environment (Extended Data Fig. 6a−d). We then tested for conjunctive cells encoding multiple modalities. We found no evidence for conjunctive encoding in LEC axons or GCs and only a small fraction (approximately 1%) in a single MEC animal. Given the rarity and limited consistency of these cases, we concluded that conjunctive coding was not a prominent feature within GCs and EC inputs. Taken together, our results suggest that the convergent LEC-mediated (predominantly olfactory) and MEC-mediated (objects, rewards and space) information streams are both incorporated and represented by GCs.

Finally, we examined in more detail the encoding of objects by MEC inputs and GCs (Fig. 5b and Extended Data Fig. 6e−g). Object-tuned MEC inputs exhibited a stronger propensity to encode for multiple objects than GCs (45% versus 9%, respectively). Indeed, we did not identify a single GC that represented objects 2−4 or 1−3 or 2−3, as observed for MEC inputs. Moreover, the arch-shaped object (2) under which the animal had to pass through was encoded by a three-fold larger GC fraction as compared to MEC axons (73% versus 23%, respectively). In accordance with the expansion theory^30,31^, these data indicate that overlapping MEC input representations are segregated at the level of the DG network into sparse, orthogonalized information streams. Such a coding mechanism may support high coding specificity of behaviorally relevant contextual details in the DG.

### Improved information content in the representation of objects and space from EC inputs to the DG

**Extended Data Fig. 7:**
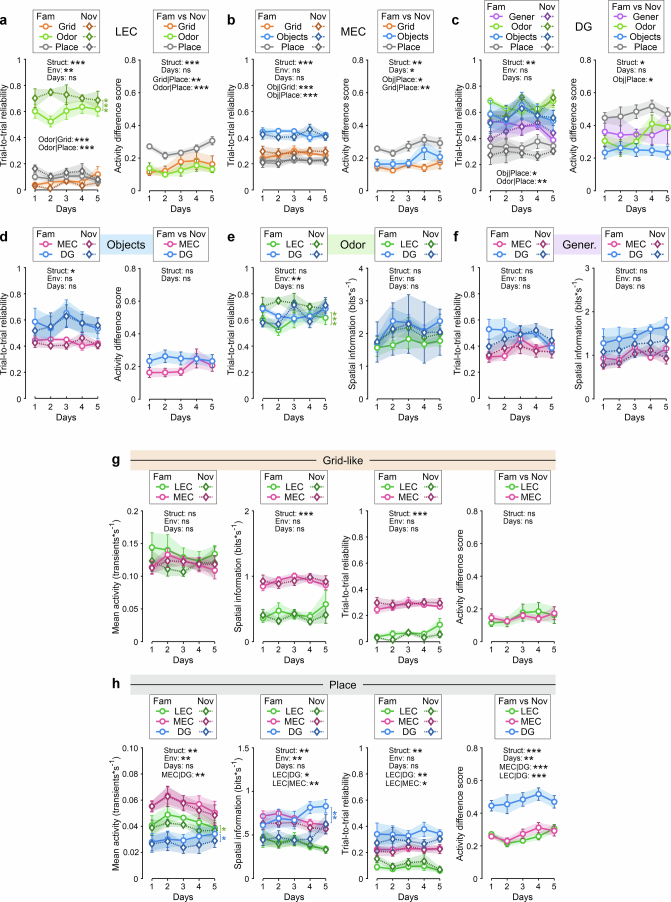
Functional characteristics of the different classes of LEC/MEC axons and GCs identified based on their activity patterns. **a-c** Trial-to-trial reliability and activity difference score (ADS) of the main types of LEC/MEC axons and GCs including LEC grid-like, odor-related activity and axons encoding for place fields (**a**), MEC grid-like, object-related activity and axons encoding for place fields (**b**), and GCs generalizing between environments, encoding for odor, objects and place fields (**c**). **d-f** Trial-to-trial reliability (left panel) and ADS or spatial information (bits*s^−1^, right panel) of objects (**d**), odor (**e**) and generalizing (**f**) axons/cells for LEC/MEC axons and GCs. **g-h** Mean activity (transients*s^−1^), spatial information (bits*s^−1^), trial-to-trial reliability and ADS of grid-like axon terminals (comparison between LEC and MEC axons, **g**) or LEC/MEC axons and GCs with place fields (**h**). **a-g** Three-way (mean activity / spatial information / trial-to-trial reliability) or two-way (ADS) repeated measures ANOVAs, Tukey’s post-hoc test. Lines with shadows indicate mean ± SEM. ns, not significant; *P < 0.05; **P < 0.01; ***P < 0.001. Black stars indicate main effects; colored stars, differences between environments within a group. For **a-h**, LEC, *n* = 5; MEC, *n* = 7; DG, *n* = 5 (datasets). For exact p values, see Supplementary Table 1.

We compared activity levels and SI of EC inputs and GC populations encoding for cues, odor or space in the open-field-like linear virtual environments (Exp2; Fig. 5c−e and Extended Data Fig. 7a−c). Odor-specific LEC LII axons exhibited markedly lower activity (Fig. 5c, left) but higher spatial tuning (Fig. 5c, right) and improved trial-by-trial reliability (Extended Data Fig. 7a, left) than LEC inputs with grid-like activity. In the MEC, grid-like axons were more active than object- associated axons (Fig. 5d, left) and showed similar SI (Fig. 5d, right) but were less reliable (Extended Data Fig. 7b, left). In GC populations, mean activity of odor, object, place or generalizing cells did not significantly vary across days (Fig. 5e, left), whereas SI was higher in odor-associated and object-associated cells and increased modestly over time in all categories (Fig. 5e, right).

We next asked whether the change in SI from EC inputs to GC outputs depended on cue modality. GCs encoding individual objects exhibited higher SI (Fig. 5f, right) and greater reliability (Extended Data Fig. 7d, left) than MEC inputs. Odor-associated LEC inputs and GCs showed similar SI (Extended Data Fig. 7e, right), high reliability (Extended Data Fig. 7e, left) and similar activity levels (Fig. 5g, left), suggesting a 1:1 transfer of olfactory information from LEC to a small fraction of GCs, although GCs showed higher ADS (Fig. 5g, right). Grid-like axons originating from the MEC displayed higher SI and reliability than those from the LEC despite similar activity levels and ADS (Extended Data Fig. 7g). Finally, GCs and MEC axons generalizing across environments showed similar activity, reliability and SI, with a tendency toward higher ADS (Fig. 5h and Extended Data Fig. 7f). Together, these results indicate that EC-to-DG information transfer is strongly modality dependent and selectively enhances SI through context-specific modulation of GC activity, a feature largely absent from EC inputs.

### While cue-, goal- and spatially tuned MEC inputs jointly encode contextual features, DG place cells define spatial coding precision

**Fig. 6:**
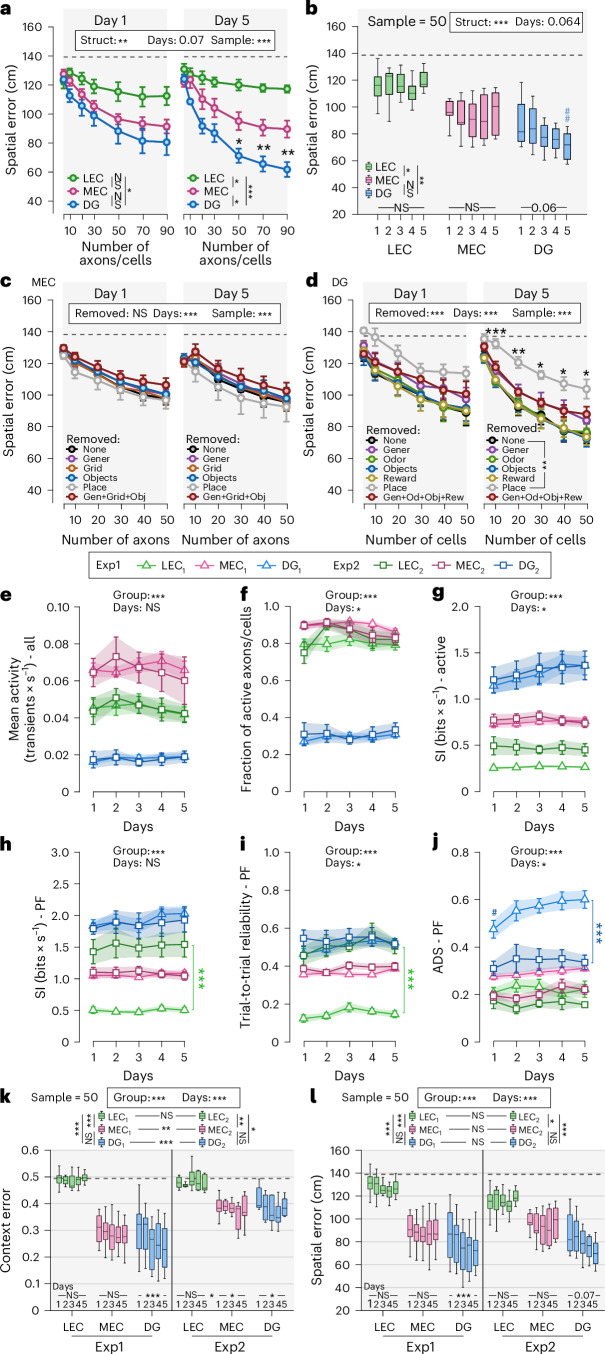
Decoding from neuronal activity in two versions of a multisensory open-field-like linear virtual environment and comparison between the decoding results of Exp1 and Exp2. **a**,**b**, Spatial decoding error as a function of the number of LEC/MEC axons or GCs simultaneously used. Panel **a** shows results depending on sample size (from 5 to 90 axons/cells), and **b** focuses on a single sample size of 50 axons/cells and the comparison between days within each structure (see also Extended Data Fig. 8). **c**,**d**, Spatial decoding error as a function of the number of MEC axons in the DG (**c**) or GCs (**d**) used simultaneously for decoding depending on the type of axons/cells removed from the total axons/cells pool. For LEC results as well as context decoding errors, see Extended Data Fig. 9. **e**−**j**, Mean activity (transients × s^−1^, **e**) and fraction of active axons/cells (>1 transients per minute, **f**) for all LEC axons (green), MEC axons (pink) and GCs (blue) recorded over five consecutive days in Exp1 (light colors, triangles) and in Exp2 (dark colors, squares) as well as SI (bits × s^−1^, **g**) of active LEC/MEC axons and active GCs and ADS (**h**), SI (bits × s^−1^, **i**) and trial-to-trial reliability (**j**) of LEC/MEC axons and GCs with PF(s) (see also Extended Data Fig. 10). **k**,**l**, Comparison of the contextual (**k**) and spatial (**l**) decoding errors observed using a fixed sample size of 50 axons/cells in the two experiments presented in this paper. Exp1 is characterized by environments with different wall and ground patterns and different reward locations (Fig. 1). Exp2 is defined as two alternate versions of the same box-like environment with different orders of cues (Fig. 4). Note that MEC axons and GCs show significantly lower contextual decoding errors in Exp2 than in Exp1. By contrast, spatial decoding errors are similar between the two experiments. **a**−**l**, Three-way (**a**,**c**,**d**) and two-way (**b**,**e**−**l**) repeated-measures ANOVAs, Tukey’s post hoc test. Lines with error bars indicate mean ± s.e.m. **a**,**c**,**d**, Data are shown here for days 1 and 5 only, but statistical tests considered the entire experiment (see also Extended Data Figs. 8 and 9). Boxes, 25th to 75th percentiles; bars, median; whiskers, 99% range. NS, not significant; ^#^/*P < 0.05; ^##^/**P < 0.01; ^###^/***P < 0.001. Black stars indicate main effects (all plots), comparisons between ‘place’ and control (‘none’) groups (**d**) and days effect within a given group (**k**,**l**); colored hashtags, difference between day 1 and day 5 (**b**). Gray dashed line shows chance level (shuffled data). For exact *P* values, see Supplementary Table 1. Gen/Gener, generalizing; Obj, object; Od, odor; Rew, reward; Struct., structure.

**Extended Data Fig. 8:**
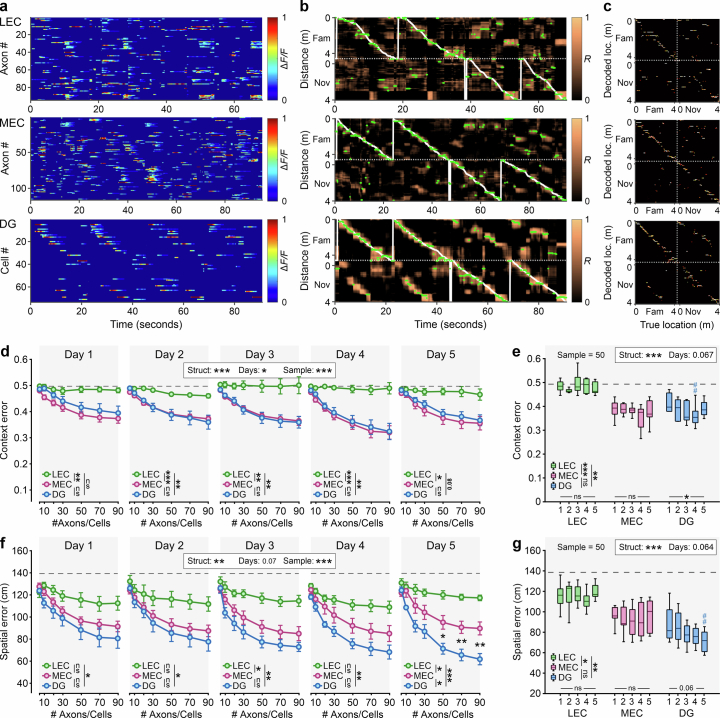
Decoding of space and context from neuronal activity between two versions of an open field-like linear virtual environment: decoding examples and 5-days results for all axons/cells together. **a-c** Decoding examples for LEC axons, top row; MEC axons, middle row; GCs, bottom row, plotted over time. **b** Decoder output. White line denotes true position of the mouse and the green dots the decoded position. **c** Confusion matrix of actual mouse location (x-axis) and maximum-likelihood decoded locations (y-axis). Upper left and lower right dotted boxes outline real location in familiar or novel environments, respectively. **d-g** Contextual (**d**) and spatial (**f**) decoding error as a function of the number of LEC/MEC axons and GCs simultaneously used for decoding and comparison between brain regions over time for a fixed sample size of 50 axons/cells (**e**, context; **g**, space). Same data as Fig. 6a-b**. d-g** Two-way (**e, g**) or three-way (**d, f**) repeated measures ANOVAs, Tukey’s post-hoc test. Lines with error bars indicate mean ± SEM. Boxes, 25th to 75th percentiles; bars, median; whiskers, 99% range. Colored hashtags, differences between day 1 and day 4 (**e**) or 5 (**g**). Grey dashed line shows chance level (shuffled data). ns, not significant; #/*P < 0.05; ##/**P < 0.01; ###/***P < 0.001. For **d-g**, LEC, *n* = 5; MEC, *n* = 7; DG, *n* = 5 (datasets). For exact p values, see Supplementary Table 1.

Using population-vector-based decoding to predict location and context, we observed that context decoding errors did not differ between MEC inputs and GCs, regardless of the day, whereas LEC inputs exhibited very low contextual decoding accuracy (Extended Data Fig. 8a−e). Conversely, spatial decoding performance of MEC inputs and GCs progressively diverged over time (similarly to Exp1), reaching maximal differences on day 5 (Fig. 6a and Extended Data Fig. 8f). Moreover, only GCs, but not MEC or LEC axons, showed significant improvements in context and space decoding with learning (Fig. 6b and Extended Data Fig. 8e,g).

**Extended Data Fig. 9:**
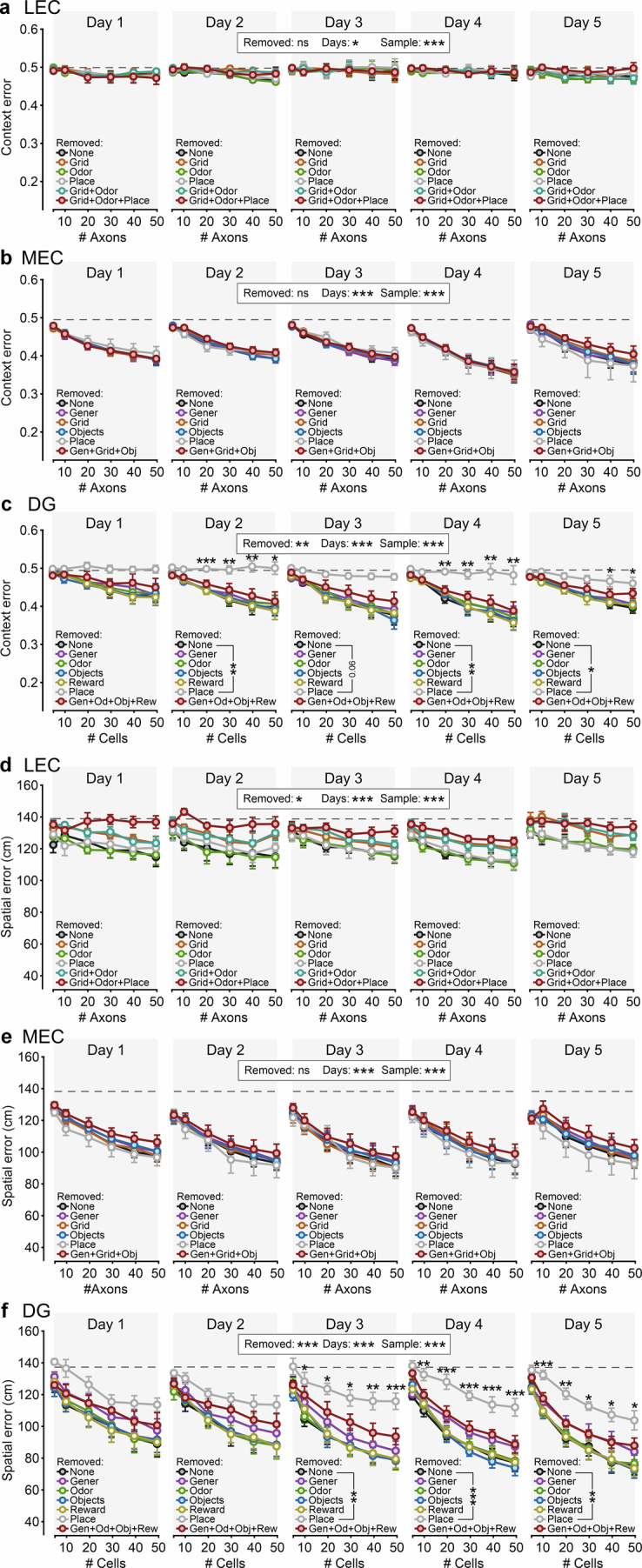
Decoding of space and context from neuronal activity between two versions of an open field-like linear virtual environment: decoding examples and 5-days results of axons/cells of specific classes. **a-c** Contextual decoding error as a function of the number of LEC axons (**a**), MEC axons (**b**) or GCs (**c**) used simultaneously for decoding. For each class (for example object encoding), axons/cells were removed in steps of 10% from the total pool of LEC/MEC axons or GCs for context decoding. **d-f** Similar to **a-c** for analysis of spatial decoding errors. Panel **e** shows the same data as Fig. 6c and panel **f** used the same data as Fig. 6d, for all days. **a-f** Three-way repeated measures ANOVAs, Tukey’s post-hoc test. Lines with error bars indicate mean ± SEM. Grey dashed line shows chance level (shuffled data); ns, not significant; *P < 0.05; **P < 0.01; ***P < 0.001. For **a,d**, LEC, *n* = 5; for **b,e**, MEC, *n* = 7; for **c,f**, DG, *n* = 5 (datasets). For exact p values, see Supplementary Table 1.

How do neuronal classes encoding PFs, grid-like patterns, objects or odors contribute to decoding performance? To address this, we selectively removed individual classes, or combinations of classes, from the axonal or cellular pools and assessed the impact on decoding performance (Fig. 6c,d and Extended Data Fig. 9). Because sample sizes were matched, removal of one class increased representation of others, potentially compensating for the loss of information. In LEC inputs, removal of grid-like, place or odor-associated axons, individually or combined, did not affect context decoding (Extended Data Fig. 9a), and, surprisingly, removal of MEC grid-like, object or PF-tuned axons had no significant effect either (Extended Data Fig. 9b). By contrast, removal of place cells from the GC population markedly decreased both context and spatial decoding, whereas removal of other classes did not (Extended Data Fig. 9c,f). Similar to context decoding, spatial decoding was not significantly influenced by the removal of individual LEC/MEC classes (Fig. 6c and Extended Data Fig. 9d,e). Thus, tuned LEC and MEC axons contribute jointly to contextual and spatial encoding, whereas DG place cells play a central role in discriminating between contexts and locations.

### Comparisons between Exp1 and Exp2 confirm a higher ability of GCs to discriminate between contexts than their MEC/LEC inputs

**Extended Data Fig. 10:**
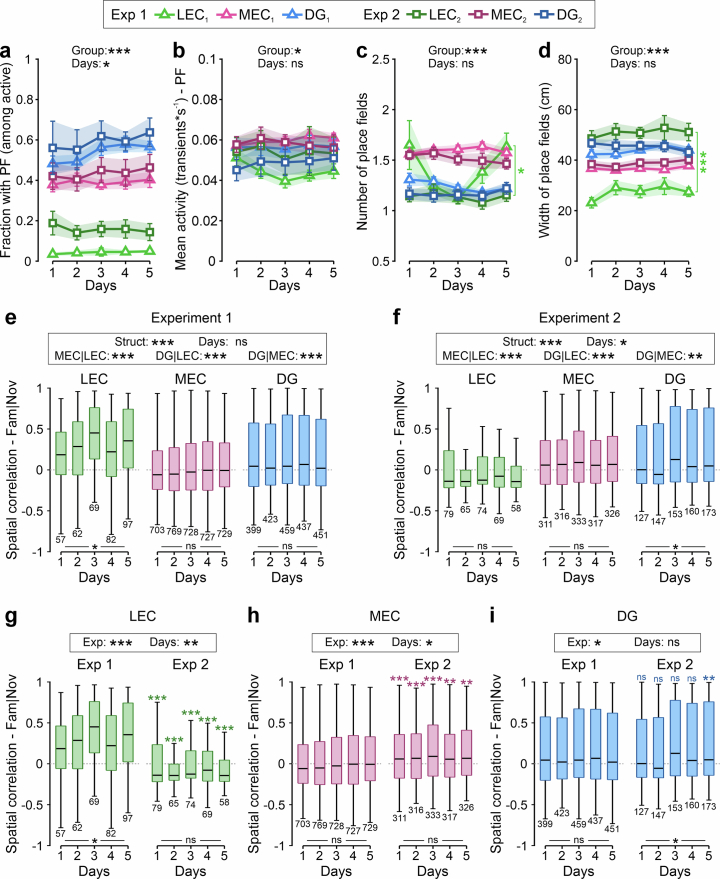
Comparisons between experiments 1 and 2 reveal similar spatial properties of MEC axons, improved spatial tuning related to odor encoding in LEC axons, and lesser discrimination between familiar and novel environments in MEC axons (and to a lower extent, in GCs) in Exp 2 than Exp 1. **a-d** Fraction of active axons/cells with one or multiple place field(s) (PF, **a**), mean activity of axons/cells with PFs (transients*s^−1^, **b**), number of PFs (**c**) and width of PFs (**d**) for LEC (green) / MEC (pink) axonal projections to the DG and GCs (blue) recorded over 5 consecutive days in Exp 1 (light colors, triangles) and Exp 2 (dark colors, squares). **e, f** Spatial correlation of LEC/MEC axons and GCs with place field(s) between familiar and novel environments in Exp 1 (**e**, same as Fig. 2o) or in Exp 2 (**f**). **g-i** Spatial correlation of LEC/MEC axons and GCs with place field(s) between the familiar and novel environment. Exp 1 compared to Exp 2 for LEC (**g**) / MEC axons (**h**) or GCs (**i**). **a-d** Two-way repeated measures ANOVAs, Tukey’s post-hoc test. **e-i** Two-way ANOVAs after alignment and ranking (Methods), Tukey’s post hoc test. Lines with shadows indicate mean ± SEM. Boxes, 25th to 75th percentiles; bars, median; whiskers, 99% range. ns, not significant; *P < 0.05; **P < 0.01; ***P < 0.001. Black stars indicate main effects; colored stars, differences between the two experiments within a given structure (LEC / MEC axons or GCs); colored stars, differences between experiments for a given day (**g-i**). For **a-d**, Exp1: LEC, *n* = 12; MEC, *n* = 15; DG, *n* = 14; Exp2: LEC, *n* = 5; MEC, *n* = 7; DG, *n* = 5 (datasets). For exact p values, see Supplementary Table 1.

The two versions of the environment in Exp2 were designed to be more similar and, therefore, harder to distinguish than the familiar and novel environments in Exp1. To test whether different degrees of environmental similarities are reflected in neuronal activity, we compared the main outcomes of both experiments (Fig. 6e−l and Extended Data Fig. 10). Mean activity, fraction of active units and SI did not differ between Exp1 and Exp2 (Fig. 6e−g). Likewise, for MEC inputs and GCs, the fraction of active units with PFs (Extended Data Fig. 10a), their activity (Extended Data Fig. 10b), SI (Fig. 6h), trial-to-trial reliability (Fig. 6i) and PF number and size (Extended Data Fig. 10c,d) were similar. By contrast, the ADS of place GCs, but not of EC inputs, was significantly higher in Exp1, indicating stronger context discrimination (Fig. 6j). MEC inputs showed higher spatial correlations in Exp2 than in Exp1, pointing to higher representational overlap in Exp2 (Extended Data Fig. 10h), an effect also observed in GCs although to a much lesser extent (Extended Data Fig. 10i). LEC odor cells strongly contributed to context discrimination in Exp2 (Extended Data Fig. 10f), showing lower PF correlations than in Exp1 (Extended Data Fig. 10e,g). Thus, even under moderate environmental dissimilarities, GCs outperform their EC inputs in context discrimination.

Finally, we compared decoding performance between the two experiments (Fig. 6k,l). Context decoding errors were significantly higher in Exp2 than in Exp1 for both MEC inputs and GCs (Fig. 6k), consistent with increased difficulty in discriminating between contexts in Exp2, whereas spatial decoding errors did not differ between experiments (Fig. 6l). Robust learning-related improvements were observed in both context and spatial decoding in Exp1, whereas such refinement was weaker in Exp2, reaching only a trend for spatial decoding. GCs showed robust gradual improvements over days in both context (Fig. 6k) and spatial (Fig. 6l) decoding in Exp1, whereas such refinement was much less pronounced in Exp2, reaching only a trend for spatial decoding (*P* = 0.07). By contrast, decoding performance of LEC and MEC inputs did not improve over time in either experiment. Thus, the learning-related changes in the tuning characteristics of GCs are reflected as progressive improvement of spatial and contextual representations, a phenomenon specific to the DG and largely independent of context similarity.

## Discussion

Using in vivo two-photon calcium imaging across five consecutive days, we characterized how information from the LEC and MEC is transmitted to the DG and progressively integrated by GC populations during learning. By longitudinally recording both LEC/MEC axonal inputs and GCs in a multisensory virtual reality paradigm comprising spatial, object, odor and auditory cues, we tracked the evolution of representations over regions and time. We found that LEC inputs predominantly convey olfactory information, whereas MEC axons provide spatial and context-related signals that emerge rapidly and remain stable. By contrast, GC representations refine gradually over days, with increasing sparsity, spatial specificity, reliability, consistency and granularity. These findings demonstrate that information transmitted from the EC to the DG is progressively transformed into a sparse, highly specific and energy-efficient code that enhances contextual decoding. We propose that such precise and stable memory traces in the DG serve as long-lasting reference frames of the global environment, which are then relayed to downstream hippocampal areas to support flexible episodic memory construction^32^.

### Improved discrimination between similar contexts in the DG compared to the EC

Pattern separation, the process by which overlapping or similar inputs are reshaped into more distinct orthogonal outputs, has long been proposed as a core DG function^9,31,33^. Exposure to a local versus global cue mismatch task showed that differences in the correlation between the two resulting neuronal representations were larger in the DG than in the EC^34^, and robust GC remapping followed subtle environmental changes despite stable EC input^33^. Our results refine this view by showing that MEC inputs, like GCs, strongly discriminate between clearly distinct environments (Exp1), as evidenced by low cross-context correlations and high decoding accuracy. However, when environments were made more similar (Exp2), MEC inputs showed reduced remapping and increased representational overlap, whereas GCs retained a higher discrimination capacity. Similarly, pronounced pattern separation in the DG, but not in CA1, was observed when replacing familiarized vertical grating patterns on the lateral walls of a virtual environment with oblique ones, despite otherwise unchanged visual cues^35^. Previous computational models demonstrated that the EC-to-DG connectivity could allow GC populations to operate as a competitive network, leading to sparse orthogonal representations^36,37^. In the present study, we experimentally confirmed this hypothesis by demonstrating that the ability to discriminate between contexts is higher in the DG than in its MEC inputs, particularly when environmental differences are subtle.

### Circuit mechanisms underlying context discrimination and generalization in the DG

A substantial fraction of MEC inputs (approximately 10% Exp1 and approximately 5% Exp2) and GCs (approximately 20% in both experiments) generalized across contexts. These subpopulations did not differ markedly in mean activity or tuning properties (Fig. 5h and Extended Data Fig. 7f). The generalizing GC population accumulated at the beginning and end of the tracks (Supplementary Fig. 5a), representing regions shared across environments. However, these cells might also encode distance relative to the start point^38^ or distance from objects or boundaries relative to the end of the track^39–41^, consistent with their absence in LEC axons. In parallel, GCs exhibiting strong context discrimination constituted the largest fraction (Fig. 2r). The mechanisms that may underlie this marked partition into two antagonistic GC populations are two-fold, presumably involving lateral inhibition provided by GABAergic cells^42,43^ and strong perforant path-mediated feedforward excitation of GC subpopulations. Disynaptic feedback inhibition of GCs by hilar mossy cells^44^ and direct dendritic inhibition by somatostatin (SOM)-expressing interneurons have been implicated in pattern separation processes^42,43,45,46^, by supporting the selection of GCs that receive the highest excitation level based on a ‘winner-takes-all’ mechanism^47^. Transcriptomic studies further suggest that Npas4-expressing GCs promote discrimination, whereas Fos-positive GCs receiving stronger MEC excitation favor generalization^42^, suggesting that specific synaptic and circuit mechanisms regulate the tradeoff between contextual discrimination and generalization in GCs^48^. Thus, dynamic engagement of these mechanisms may regulate the balance between generalization and discrimination.

Alternatively, the observed differences in the fraction of discriminating versus generalizing GCs between Exp1 and Exp2 could predominantly arise from GCs being tuned to specific visual inputs. In Exp1, environments differed broadly in visual textures, which may enhance context discrimination, whereas, in Exp2, the environments were visually sparse except near the objects, possibly leading to weaker discrimination and more generalization at visually matched locations. Such a view-based perspective suggests that differential visual tuning, rather than competitive circuit mechanisms alone, might underlie the balance between generalization and discrimination in GCs and could also explain the apparent overrepresentation of object 2.

### Slow emergence of a novel spatial code in the DG

In contrast to MEC/LEC inputs, GC spatial representations of the novel environment progressively improved over days (Figs. 3d−g and 6a,b), with increasing number of active GCs and augmenting SI over the initial 3−4 days, eventually reaching levels similar to cells representing the familiar environment (Fig. 2c−e). DG representations have been shown to require time to fully stabilize^49^, but the cellular and circuit mechanisms underlying these gradual changes remain unclear. The high intrinsic excitability threshold of GCs (due to their more negative membrane resting potential of approximately −80 mV^50^) compared to CA1−3 PCs (approximately −70 mV^51^), their lack of recurrent synapses and the high-frequency induction paradigms needed to evoke long-lasting plasticity at EC−GC synapses in vitro^52^ collectively favor a slow timecourse in the recruitment of GCs. Moreover, SOM cells in the DG discharge at higher rates in novel environments^43^, suggesting that lateral dendritic inhibition of EC inputs may temporally restrict their excitability. Alternatively, the transformation of entorhinal inputs by GCs could also arise from a combination of intrinsic cellular properties and synaptic nonlinearities. The very negative resting membrane potential of GCs may bias them toward responding preferentially to temporally contiguous bursts of EC inputs, such that synaptic input integration might be more accurately reflected by the tuning of activity bursts in GCs rather than general temporally unrelated input profiles. Future studies should consider how burst dynamics and synaptic nonlinearities interact with competitive network mechanisms, such as lateral dendritic inhibition, to shape the transformation of inputs in the DG.

The slow emergence of contextual maps in the DG contrasts with the rapid formation of place cells in CA1 (ref. ^53^). However, recent population imaging studies have revealed gradual formation of orthogonalized cognitive maps in CA1 that capture the structure of the learned task over timescales similar to those that we observed for novel DG spatial maps^54^. Thus, slow DG refinement might set the pace for the progressive emergence of task-relevant representations in downstream hippocampal regions. In this proposed model, the DG provides a stable, multimodal reference frame emphasizing persistent context features, which are then combined in CA1 with rapidly changing task-related information^5,55^.

### Higher granularity of object representations from MEC to DG

Cells encoding vector relationships to objects were first reported^40^ and later characterized^41^ in CA1 and CA3. Object-vector cells were subsequently identified in the MEC^39^ and defined as neurons that specifically discharge at given distances and directions from a spatially confined object^39^. Incidentally, object-vector cells in the MEC respond to two-dimensional objects, making them a potential source of visual anchoring points under virtual reality paradigms^56^. Using tetrodes recordings, representations of objects in the DG have first been found in mice running on a treadmill belt with fixed landmarks^49^. GCs appeared to encode conjunctions of objects and spatial information and mostly display single PFs, whereas mossy cells preferentially represented object locations and showed multiple fields. This finding was confirmed in freely moving mice foraging for rewards^57^; however, most recorded cells were mossy cells exhibiting multiple firing fields at fixed distances and directions from objects. Our axonal recordings confirm that many MEC inputs encode multiple objects, whereas object-tuned GCs typically anchor their activity to single objects (Fig. 5a,b). Divergent MEC inputs distributed over large GC populations^58^, together with the fast timecourse of excitatory postsynaptic potentials (EPSPs) at distal GC dendrites supporting synaptic summation over narrow time windows^52^, likely promote input-specific information processing, enhanced specificity and discrimination of objects by GCs. This enhanced granularity from MEC to DG on the level of objects representation also contrasts with the more direct and transparent transfer of odor information from LEC to DG (Fig. 5g and Extended Data Fig. 7e), indicating that, depending on input pathway and sensory modality, the neural code of GCs does not always markedly differ from cortical representation.

### Area-specific information content of MEC and LEC projections to the DG

The projections from the MEC to CA1−3 and DG are highly target area specific^17,24^. MEC superficial layers contain two main cell types, stellate and pyramidal cells, differing in their morphology, intrinsic properties and neurochemical marker contents^59^. In mice, pyramidal cells primarily project to the ipsilateral CA1 and the contralateral MEC^60^, whereas axons of stellate cells mostly terminate in the DG but not in CA3 (refs. ^12,24^). Finally, MEC axonal projections to the DG are restricted to the ipsilateral hemisphere, whereas those targeting CA1 are running bilaterally^24^. Thus, each hippocampal subfield receives specific allosteric information through distinct EC-to-hippocampus pathways. By imaging MEC inputs in CA1, CA3 and the DG, we previously demonstrated differences in the mean activity, spatial tuning characteristics and reliability in the representation of familiar environments, pointing to a high target area specificity in the transmitted contextual content^12^. A projection specificity has also been observed for LEC LII fan cells, which project to the DG, whereas LIII pyramidal cells mainly target CA1 and the subiculum^13^. Consistent with previous LEC studies^6,11^, we observed that spatially tuned LEC inputs are very rare in the DG (Fig. 2d,k). Moreover, LEC axonal activity was markedly more correlated between contexts than MEC or DG responses (Fig. 2p). Superficial layer cells in the LEC have been shown to discharge in response to non-spatial features such as odors^16,61^, goals^11^ and objects^62^. Local versus global cues mismatch tasks further indicated that LEC cells are more controlled by local rather than global cues^34^. Our data appear to partially deviate from those findings, as odors, but not two-dimensional cues, were the main determinant controlling LEC input activity in the DG (Fig. 5a). One potential explanation to this apparent discrepancy might be that, similar to the MEC^12^, LII/LIII LEC cells project to the DG, CA1 and CA3 in a target area-specific manner, transmitting different environmental information. LEC spatial-coding and object-coding properties vary along its anterio-posterior axis^62^, and LEC LII fan cells do not respond to novel objects nor do they support context-related object recognition, as opposed to superficial LEC layers taken as a whole^62^. Finally, two-photon calcium imaging of LEC axons in CA1 of mice navigating virtual realities revealed that their activity and association with reward locations did not increase after adding olfactory and auditory cues to goal sites^11^. Thus, LEC projections to CA1 transmit signals accounting for the association between reward and location but virtually no olfactory information, whereas this seems to be the opposite for LEC-to-DG axons. This dichotomy is in line with the observed overrepresentation of reward locations by CA1 place cells as compared to the rest of the environment^4,5,63^, a phenomenon not observed in the DG (Fig. 2m; refs. ^8,12,26^). These results have important implications for studies investigating the information transfer between EC and hippocampus as they underscore the importance of considering which one of the parallel pathways (characterized by the specific projection patterns of distinct EC cell populations to the hippocampal areas) they follow.

Taken together, this work experimentally validates the longstanding view that LEC and MEC mediate contextual and spatial information, respectively, and that DG GCs are more sensitive to small environmental changes than their EC inputs. Notably, it challenges the classical view that LEC is providing object information to the whole hippocampus: in contrast to CA1, in the DG, object-related information is provided to GCs by the MEC. It also demonstrates that the emergence of stable LEC, MEC and DG representations follow different temporal dynamics: novel representations emerge very rapidly in LEC/MEC inputs (as early as day 1) and slowly in GCs (progressive improvement over the 5 days of experiment). Finally, it shows how incoming spatial and non-spatial information from the EC is integrated and represented on the level of GC populations. Future research should investigate the molecular mechanisms underlying the slow integration of EC-mediated spatial and non-spatial information in GCs as well as the influence of neuromodulatory inputs on the slow GC recruitment and their role in shaping EC−DG transformations.

## Methods

### Experimental model and subject details

#### Animal experiments

All experiments involving animals were carried out according to national and institutional guidelines and approved by the ‘Tierversuchskommission’ of the Regierungspräsidium Freiburg (license no. G22/088) in accordance with national legislation. We used a total of 40 C57BL/6J wild-type mice aged 9−12 weeks at the beginning of the experiments. In Exp1, six mice were injected in the LEC, 10 in the MEC and seven in the DG. In Exp2, five mice were injected in the LEC, seven in the MEC and five in the DG. Exp1 comprised 10 males and 13 females, and Exp2 comprised seven males and 10 females, randomly distributed across experimental groups. All animals were implanted with a chronical imaging window over the hippocampus. In Exp1, most animals were recorded twice using two different novel environments to obtain two independent datasets per animal (for a total of 41 datasets; 12 LEC, 15 MEC and 14 DG), allowing us to reduce the number of animals. Mice were housed on a 12-hour light/dark cycle in groups of 2−3 mice in a room maintained at a temperature of 21 °C (±1 °C) and relative humidity of 55% (±10%).

### Methods details

#### Virus injections and headplate implantation

All surgical procedures were performed in a stereotactic apparatus (Kopf Instruments) under anesthesia with 1.5–2% isoflurane and analgesia using 0.1 mg kg^−1^ buprenorphine. An eye lubricant ointment (Bepanthen; Bayer) was applied to protect corneal membranes during surgeries. The skin was first disinfected twice with alternating solutions of 70% ethanol and povidone-iodine (Betadine; Avrio Health L.P.) before surgical incision. In LEC mice, a small craniotomy (diameter 0.5−1 mm) was made over the LEC (A/P +0.63 mm from the lambdoid suture, M/L −0.33 mm from lateral crest of parietal bone). Dura was carefully pierced with a needle in the center of the craniotomy, and a glass micropipette was lowered at D/V −2.60 mm below the brain surface, where 100 nl of AAV1-hSynapsin1-axon-GCaMP6s (titer 1 × 10^12^ viral genomes per milliliter (vg/ml); Addgene plasmid no. 111262 (ref. ^64^)) was slowly injected into the LEC. In MEC animals, a small craniotomy (diameter 0.5−1 mm) was made over the MEC (A/P −0.73 mm from the lambdoid suture, M/L 3.25 mm from lambda). Dura was carefully pierced with a needle in the center of the craniotomy; a glass micropipette was lowered at an angle of −9° (in the antero-posterior axis); and 100 nl of AAV1-hSynapsin1-axon-GCaMP6s (titer 1 × 10^12^ vg/ml; Addgene plasmid no. 111262 (ref. ^64^)) was slowly injected while progressively moving the tip of the needle from D/V −1.70 mm to −1.40 mm (from brain surface) in order to allow infection of layer 2/3 over the ventro-dorsal extend of the dorsal MEC. In DG GC animals, a small craniotomy (diameter 0.5−1 mm) was made over the hippocampus (A/P −2.0 mm, M/L ±1.4 mm from bregma). Dura was carefully pierced with a needle in the center of the craniotomy, and a glass micropipette was lowered at D/V −1.80 mm below the brain surface, where 400 nl of AAV1.Syn.GCaMP6s.WPRE.SV40 (titer 1 × 10^12^ vg/ml; Addgene plasmid no. 100843 (ref. ^65^)) was slowly injected in the DG. In all animals, the target volume was slowly injected over approximately 2 minutes, and the glass micropipette was further left in situ for 10 minutes to ensure complete diffusion of the viral vector in the parenchyma. During the same surgical procedure, mice were implanted with a stainless steel headplate (25 × 10 × 0.8 mm with 8-mm-wide central aperture) installed horizontally over the hippocampus, centered on A/P = −2.0 mm and M/L = ±1.8 mm from bregma and secured with dental cement (Super-Bond Universal Polymer Radiopaque, Catalyst V and Quick Monomer; Sun Medical). Postoperative analgesic treatment consisted of carprofen administration (5 mg kg^−1^ of body weight) provided during 3 days after surgery. Mice were allowed to recover from surgery for at least 5 days before any further experiment.

#### Imaging window implantation

During a second surgery taking place at least 5 days after the first one (see above), an imaging window was implanted. Using the same anesthesia/analgesia protocol as described above, a craniotomy (diameter 3 mm) was drilled at A/P −2.0 mm, M/L ±1.8 mm. Under continuous irrigation with chilled saline, part of the somatosensory cortex and posterior parietal association cortex located above the hippocampus was progressively aspirated until the external capsule was exposed. The outer part of the external capsule was then gently peeled away using fine forceps, leaving the inner capsule and the hippocampus optically accessible yet undamaged. The imaging window implant consisted of a 3-mm-wide coverslip (CS-3R; Warner Instruments) glued to the bottom of a stainless steel cannula (3-mm diameter, 1.3-mm height). This window was gently lowered into the craniotomy using forceps until the coverslip was sitting on the external capsule. The implant was then fixed to the surrounding skull using cyanoacrylate. Mice were allowed to recover from window implantation for at least 4 days before any further experiment.

#### Virtual environment setup

As previously described^8,12,66,67^, our custom virtual environment setup consisted of an air-supported polystyrene ball (20-cm diameter) attached on one side with a small metal axle (restraining the ball motion to the forward–backward direction). Ball movement was monitored using an optical sensor (G-500; Logitech) and translated into forward motion inside the virtual environment. The forward gain was set such that 4 m of distance travelled along the circumference of the ball equaled one full traversal of the linear track. When the mouse reached the end of the track, screens were blanked for 4−10 seconds before the mouse was ‘teleported’ back to the start of the linear track. The virtual environment was displayed on four TFT monitors (19″ screen diagonal; Dell) arranged in a hexagonal arc around the mouse and placed approximately 25 cm away from the head of the animal, thereby covering approximately 260° of the horizontal visual field and approximately 60° of the vertical visual field of the mouse. Virtual environments were created and simulated using the open-source three-dimensional rendering software Blender 2.79b (available at https://www.blender.org/). The seven (five in Exp1, two in Exp2) different environments used in the present work consisted of distinct arrangements of textured walls, floors and three-dimensional rendered objects placed along the track sides. All environments contain two rewards of which the locations differed between environments (Fig. 1 and Supplementary Fig. 1). At rewarded sites, mice received 2 µl of soy milk dispensed through a spout in front of the animal. In Exp2, one olfactory stimulus and one auditory stimulus were delivered at specific locations along the track. The coconut odor was prepared by warming up coconut oil until it became liquid and applied on a cotton swab tip. The coconut-loaded piece of cotton was then put into one of the four odor wells available on the custom-designed odor dispenser in order to deliver the olfactory stimulus as the head-fixed animal navigated in a virtual reality. This device consists of four independent channels of which the opening is controlled by solenoid valves (Festo MHE2-M1H-3/2G-M7-K) coupled to a custom Arduino board. All channels converge into a single delivery cannula positioned 20 cm away from the mouse and pointing at its snout. By switching between channels, a constant airflow is maintained, allowing for rapid delivery as well as fast removal of the previous scent. In practice, the dispenser was configured to continuously deliver odor-free air (default position: unscented channel 1 open, channel 2 containing the odor closed) and was briefly switched to the coconut-loaded channel (channel 2 open, channel 1 closed) for 1 second to deliver the odor when the animal reaches the corresponding position along the track. The constant air flow and short tubing ensured that the delay in odor delivery was ≤0.2 seconds, and the coconut odor was rapidly distributed and expulsed from the mouse vicinity after delivery. The auditory stimulus was a 6-kHz pure tone played for a duration of 1 second at a level of 80 dB sound pressure level (SPL).

#### Behavioral training

Five to 7 days after headplate implantation, mice were allowed to explore a first (hereafter called ‘familiar’) virtual environment for 10−30 minutes daily, with gradually increasing timespans over days. Once mice showed evidence of habituation to this task (that is, appropriate position on the ball and consistent voluntary running, usually after 5−10 days of training), food scheduling was initiated with a goal of approximately 85% of the ad libitum body weight. Training in the familiar environment was maintained for 30–60 minutes daily until consistent reward licking and voluntary running were observed (that is, after at least 12−15 days of exposure to the familiar environment, before any imaging session started). In Exp1, the familiar environment was one of the five environments presented in Supplementary Fig. 1 (pseudo-randomly attributed to each mouse before the start of the experiment). The novel environment(s) were picked among the four remaining environments. In Exp2, familiar and novel environments were fixed, alternate versions of the same environment, as presented in Fig. 4 and Extended Data Fig. 5.

#### Behavioral paradigm for imaging sessions

From the first day of imaging, mice were introduced to a novel context, which had the same dimensions as the familiar one but with different visual cues, floor and wall textures (Supplementary Fig. 1). For each imaging session, mice alternatingly ran in the familiar environment and in one novel environment for a total of 30 runs per day. Runs were grouped by blocks of five laps for each environment (starting with the familiar one), for a total of three blocks and 15 recordings for each environment. This recording procedure was repeated over five consecutive days, during which the same exact field of view (and, therefore, the same population of axons/cells) was imaged (Fig. 1c). In many of the mice, the visible area under the imaging window was sufficiently large to allow for the selection of several imaging fields of view that contained different populations of axon terminals or GCs. In Exp1, we, therefore, replicated this procedure a second time for most of the animals, exposing them to a second novel environment. The familiar environment remained always the same for a given animal. In this manner, we acquired, in total, 41 datasets of 5 days for each type of axonal projections and cells (12 LEC axons, 15 MEC axons, 14 GCs) in Exp1 and 17 datasets (five LEC axons, seven MEC axons, five GCs) in Exp2. Grand totals of LEC axon terminals imaged were of 1,310 and 1,389 MEC axon terminals and 2,027 GCs in Exp1 (mean per session ± s.e.m.: 109.2 ± 9.8 LEC axons; 92.6 ± 6.5 MEC axons; 144.8 ± 11.6 GCs) and in Exp2: 431 LEC axons, 632 MEC axons and 650 GCs (mean per session ± s.e.m.:, 86.2 ± 8.3 LEC axons; 90.3 ± 8.4 MEC axons; 130.0 ± 14.4 GCs).

#### Reward-related licking behavior

Licking by mice was monitored using an infrared optical lick detector placed in front of the metal lick spout dispensing the reward. For some of the recording sessions, no lick data were acquired. In total, lick data were obtained from eight LEC datasets, 13 MEC datasets and 11 GCs datasets in Exp1, respectively, and from two, seven and five datasets in Exp2, respectively. The reward zones were defined as the six bins (that is, 30 cm) around the center of the reward sites. The remaining of the track was considered as outside of the reward zones. Thus, the ratio of licking between inside and outside of the reward zones was computed as the mean lick rate in the reward zones divided by the mean lick rate on the remaining track (Supplementary Fig. 2).

#### In vivo two-photon calcium imaging

In vivo calcium imaging was performed using a resonant/galvo high-speed laser scanning two-photon microscope (Neurolabware) through a ×16 objective (0.8 numerical aperture, 3 mm working distance; Nikon) with a frame rate of 15.5 Hz and using a single plane for imaging. GCaMP6s was excited at 930 nm with a femtosecond-pulsed two-photon laser (Mai Tai DeepSee; Spectra-Physics). To block ambient light from reaching the photodetectors, the animal’s headplate was attached to the bottom of an opaque imaging chamber before each experiment, and the mouse was then affixed to the virtual environment setup using this head chamber. A ring of black foam rubber was placed between the imaging chamber and the microscope objective, and a metallic collar surrounding the objective was sitting on the imaging chamber, blocking any remaining stray light. Laser power and photomultiplier detectors (H11706-40 GaAsP; Hamamatsu) were compensated appropriately for each imaging session, ensuring consistent recording conditions. To help find the exact same field of view over multiple days, we used Rhodamine B injected intraperitoneally, which offers several advantages. (1) The labeling of blood vessels in the brain allows the same imaging region (and axons/cells) to be relocated during each successive daily imaging session. (2) The Rhodamine B fluorescence signal is constant and lasts, unlike GCaMP signals, which are dependent on neuronal activity. (3) The Rhodamine fluorescence has a different wavelength than the GCaMP signal that we record to measure neuronal activity and, therefore, does not interfere with the GCaMP (Fig. 1g−I and Extended Data Fig. 2). We injected (intraperitoneally) 100 µg (for a 25-g mouse) of Rhodamine B dextran (diluted in 0.1 ml of PBS) once daily during the experimental period. This corresponds to 4 µg g^−1^ (or 4 mg kg^−1^) of the body weight of the mouse. Rhodamine B is rapidly eliminated from the rodent body^68^; mice can be imaged immediately after injection; and the dye is excreted in the urine (which turns pink) after about 2 hours. Furthermore, Rhodamine B has already been successfully used for in vivo two-photon microscopy^69–71^.

#### Histology

At the end of the experiments, mice were deeply anaesthetized using a mixture of ketamine/xylazine (Sigma Aldrich) and then intracardially perfused with 0.1 M PBS for 5 minutes followed by 4% paraformaldehyde (PFA) in PBS for 10 minutes. Brains were further immersed in 4% PFA for 3 hours and then kept in PBS until they were cut into 80-µm-thick sagittal sections containing the EC and the hippocampus (usually the day after). Slices were counterstained with DAPI and mounted in Mowiol. Image stacks of GCaMP6s and DAPI fluorescence were acquired with a confocal microscope (LSM 710; Zeiss). In LEC-injected and MEC-injected animals, fluorescence confocal images were used to confirm that the GCaMP6s infection was centered on layers 2/3 and restricted to the structure (Fig. 1d−f).

### Quantification and statistical analysis

#### Calcium imaging data processing and ROI extraction

The processing of all raw calcium data was done using the Python-based toolbox Suite2p (v.0.14.4), a free automated pipeline for processing two-photon calcium imaging recordings (available at https://github.com/Mouseland/suite2p). In brief, Suite2p first aligns all frames of a calcium movie using two-dimensional rigid registration based on regularized phase correlation, subpixel interpolation and kriging^72,73^. This toolbox then allows visual inspection of the registered movie. Only datasets in which consistent alignment over the entire course of the experiment (that is, 5 days of recording) was achieved were kept for further processing. Suite2p performs automated axons/cells detection and neuropil correction by computing a low-dimensional decomposition of the data, which is used to run a clustering algorithm that finds ROIs based on the correlation of the pixels inside them. All ROIs were manually curated to ensure the most accurate selection of axons/cells, and care was taken to verify that segmented entities were clearly visible throughout the entire experiment, which is particularly relevant for tiny structures such as axonal projections (Extended Data Fig. 2 and Supplementary Fig. 3). GCs were mainly identified based on size and location, as the cells can be disambiguated from other cell types by their small soma size and their location in the GC layer. Neurons with unusually large somata or locations within the hilus were discarded. Finally, the frequency and shape of calcium transients were also used to discard any putative interneuron.

Data were analyzed using MATLAB 2024b (MathWorks). Significant calcium transients were identified as previously described^66,74^. This approach has been used in numerous hippocampal in vivo calcium imaging studies by others^67,75,76^ and in our laboratory^8,12,26^. We restricted our analyses to periods with a running speed of at least 5 cm s^−1^. In brief, calcium traces were corrected for slow changes in fluorescence by subtracting the 8th percentile value of the fluorescence value distribution in a window of 20 seconds around each timepoint from the raw fluorescence trace. We obtained an initial estimate on baseline fluorescence by calculating the mean and standard deviation of all points of the fluorescence signal that did not exceed 1.9 s.d. of the total signal. We then divided the raw fluorescence trace by this value to obtain the Δ*F*/*F* trace. This trace was used to determine the parameters for transient detection that yielded a false-positive rate (defined as the ratio of negative to positive oriented transients) of less than 5% and extracted all significant transients from the raw Δ*F*/*F* trace^66^. Definitive values for baseline fluorescence and baseline standard deviation were calculated from all points of the trace that did not contain significant transients. A transient mask was created, and, for further analysis, all values of this Δ*F*/*F* trace that did not contain significant calcium transients were set to zero^66^ in order to improve the signal-to-noise ratio. Using this method, Δ*F*/*F* is expressed in units of standard deviation (the standard deviation of the baseline fluorescence).

#### Activity differences and spatial information

Activity rate difference scores were calculated using the following formula: | (activity_EnvA_ – activity_EnvB_) | / (activity_EnvA_ + activity_EnvB_). To measure the SI content, we adapted a common method of SI assessment^77^ from calcium imaging data. The average calcium activity (mean Δ*F*/*F*) was computed for each 5-cm-wide bin along the linear track and used as an approximation for the neurons’ average firing rate in that location. As previously described^8,12^, SI was calculated as $\mathrm{SI}={\sum }_{i=1}^{N}{\lambda }_{i}\mathrm{ln}\frac{{\lambda }_{i}}{\lambda }{p}_{i}$ in which ${\lambda }_{i}$ and ${p}_{i}$ are the average calcium activity and fraction of time spent in the *i*th bin, respectively; $\lambda$ is the overall calcium activity averaged over the entire track; and $N$ is the number of bins on the track (80 bins in total). Therefore, SI content is inferred from differences in the calcium activity and expressed as bits × s^−1^. For each cell, significant SI was assessed by shuffling *y* traces (position of the animal along the track) of the original dataset and computing the SI score of the resulting shuffled dataset. This procedure was repeated 1,000 times, and the *P* value was determined as the fraction of shuffled datasets in which the SI score was higher than the SI score of the original dataset. SI was considered significant if *P* < 0.05.

#### Speed modulation

Speed information was computed in a very similar way as SI but using the average calcium activity computed for speed bins as an approximation for the neurons’ average firing rate depending on speed. A speed score was defined as the correlation coefficient between activity (Δ*F*/*F*, significant transients only) and running speed. Finally, for each axon/cell, a speed class was attributed by shuffling *y* traces (position of the animal along the track) of the original dataset and computing the speed score of the resulting shuffled dataset. This procedure was repeated 1,000 times, and the *P* value was determined as the fraction of shuffled datasets in which the speed score was higher than the speed score of the original dataset. If this *P* value was less than 0.05, the speed score of this axon/cell was compared to the shuffled data. If this speed score was higher than or equal to the 99th percentile of the shuffled data, the axon/cell was considered positively speed modulated (speed class ‘+’). If its speed score was lower than or equal to the 1st percentile of shuffled data, it was considered negatively speed modulated (speed class ‘−‘). If none of these conditions was fulfilled, the axon/cell was considered not significantly speed modulated (speed class ‘NS’; see also Extended Data Fig. 1).

#### PF identification

PFs were identified according to published methods^8,12,26,66,67^. In brief, the mean Δ*F*/*F* was computed from significant calcium transients for each 5-cm-wide bin along the linear track (80 bins), and this mean fluorescence over distance was then smoothed by averaging over the three adjacent points for each bin. Potential PFs were initially identified as contiguous regions of this Δ*F*/*F* over distance plot in which all of the points were greater than 25% of the difference between the bin with the highest Δ*F*/*F* value and the baseline value (mean of the lowest 20 out of 80 bins’ Δ*F*/*F* values). In addition, the candidate PFs had to fulfill the following criteria: (1) the width of the potential field had to be of at least three bins (corresponding to 15-cm running distance); (2) the mean Δ*F*/*F* value inside the field had to be at least seven times the mean of the Δ*F*/*F* value outside the field; and (3) significant calcium transients had to be present at least 20% of the time in which the mouse was moving in the field. Potential PFs that fulfilled these criteria were accepted if their *P* value from bootstrapping exceeded 0.05. For bootstrapping, the Δ*F*/*F* trace for each experiment was broken into segments of 50 consecutive imaging frames and randomly shuffled, and this was performed 1,000 times. Then, the PF detection procedure described above was performed on each of the shuffled Δ*F*/*F* traces, and the *P* value of the PF was defined as the number of these randomly shuffled traces on which a PF was detected according to the outlined criteria divided by the number of shuffles (that is, 1,000).

#### Spatial correlation (similarity between contexts/remapping) and trial-to-trial reliability

To assess the similarity of a place cell spatial representation in different environments, we calculated the mean Δ*F*/*F* value for each of the 80 bins on the track, based on all significant calcium transients (activity map) for each cell and environment. As previously described^12,26^, each recording session consisted of three blocks of five runs in each environment (for a total of six blocks and 30 runs). The similarity of PFs between contexts (remapping) was quantified as the correlation of mean activity maps for all runs in familiar and novel environments. The trial-to-trial reliability was computed by calculating the pairwise cross-correlations between the calcium signals of all individual runs in one session on the same track and then averaging the obtained values for each cell.

#### Identification of generalizing, grid-like, object(s)-associated, reward-associated, odor-associated and sound-associated axons/cells

To ensure accurate classification of axons/cells, we designed a classifier based on several criteria for each type (see details below). Each active axon/cell was first checked for generalization, then for odor, reward, sound, single-object or multi-object spatial activity association and, finally, for grid-like patterns. This order was kept for all studied axons/cells. Remaining axons/cells encoding for PFs were assigned to the ‘place’ class. As this classifier was designed using criteria that do not allow cells to be identified in more than one category (due to unique restrictions in the spatial bins along the track; see also Extended Data Fig. 5a), each cell/axon could belong to only one category.

#### Generalizing category

Generalizing axons/cells were identified based on the similarity of their spatial activity between the two environments using the following criteria: (1) having one or more PF(s) in both familiar and novel environments and (2) showing a cross-correlation value of place maps (Pearsonʼs correlation coefficient) greater than or equal to 0.6 between familiar and novel environments.

#### Odor/reward/sound category

These axons/cells were identified using the following criteria: (1) having one or more PF(s) in both familiar and novel environments; (2) having no more than two PFs per environment; (3) having a PF in familiar environment as well as a PF in novel environment within 40 cm after the odor/reward/sound onset (eight spatial bins starting with the bin of the stimulus); (4) when shifting the place field activity in the two environments in opposite directions, peak cross-correlation must be located within an offset window reflecting the distance between the stimulus’ locations in the two environments (+0.7 m to +1.3 m for odor and reward, −1.6 m to −2.4 m for sound); and (5) this peak cross-correlation value must be greater than or equal to 0.6.

#### Single-object category

Axons/cells encoding single objects were identified using the following criteria: (1) having one or more PF(s) in both familiar and novel environments; (2) having no more than two PFs per environment; (3) having a PF in familiar environment as well as a PF in novel environment within 60 cm around the object (12 spatial bins, starting three bins before the object bin); (4) when shifting the spatial activity in the two environments against each other, peak cross-correlation must be located within an offset window reflecting the distance between the object’s locations in the two environments (0.8 m to 1.2 m for 1-m distance, 1.7 m to 2.3 m for 2-m distance, 2.6 m to 3.4 m for 3-m distance); and (5) this peak cross-correlation value must be greater than or equal to 0.6.

#### Multi-objects category

Axons/cells encoding multiple objects were identified using the following criteria: (1) having one or more PS(s) in both familiar and novel environments; (2) in each environment, the peak of activity of each PF was tested for location within 60 cm around each object; (3) in each environment, the objects represented are identified as the one(s) of which the cumulated frequency of identification reaches 0.7 or more; (4) two or more objects have to be detected as represented by the cell/axon in each environment; (5) the same number of represented objects has to be detected in both environments; and (6) the same objects have to be represented by the axon/cell in both environments.

#### Grid-like category

To identify grid-like patterns, we used an approach similar to ref. ^78^ and ref. ^79^ that we employed previously^12^. This classifier identifies grid patterns based on the following criteria: (1) a grid-like axon/cell must have at least two spatial fields in an environment; (2) for an environment of length *L* (here, 4 m) and a mean field width *W*, the activity of the axon/cell must have a number of transitions between in-fields and out-of-fields periods larger than *L* / 5 × *W*; (3) the width of the largest field must be smaller than 5 × *W*; (4) at least 30% of the bins must be assigned to either in-fields or out-of-fields periods; and (5) the ratio between the mean activity (Δ*F*/*F*) during in-fields and out-of-fields periods must be larger than 2.

#### Conjunctive encoding

To assess the possible presence of conjunctive encoding, we searched for axons/cells belonging to more than one non-overlapping category simultaneously. Multi-object cells were excluded from this analysis. Conjunctive axons/cells were identified based on their reliable tuning to at least two different modalities (odor, sound, reward or object) across both contexts. The criteria were as follows: (1) having two or more PFs in both familiar and novel environments; (2) the peak activity of each PF is located within 60 cm around an object or 40 cm after odor, sound or reward delivery; (3) two or more different sensory cues are represented in each environment; (4) the same number of cues are represented in both environments; and (5) the same cues are represented in both environments. Using this approach, we did not identify any conjunctive cells in LEC axons or DG GCs, and only four axons in one MEC animal (approximately 1% of axons in that dataset) showed putative conjunctive coding (odor combined with object or sound). Based on the very low fraction and the fact that these observations were restricted to a single animal, we concluded to a lack of evidence for conjunctive coding in the EC-to-DG circuitry.

#### Population-vector-based decoding

As described previously^12,26^, to decode position and context from neuronal activity data, we first split every dataset in two interleaved halves of template and testing runs, respectively. Templates for population-vector-based decoding were then generated using the template runs by calculating the mean activity for each 5-cm bin on familiar and novel linear tracks. Neuronal activity from the testing data was used to calculate population activity vectors for each 100-ms bin, and we computed the Pearsonʼs correlation value for each of those population vectors with the template population vectors for each position. The most likely decoded location was then determined as the spatial bin that had the highest correlation with the population activity at a given time^25^. Context error for each time bin was either 0 if the decoded location was in the correct environment or 1 otherwise. The mean context error was then obtained by averaging over all time bins. To obtain the mean spatial error, we calculated the absolute distance between the most likely decoded spatial location (irrespective of the decoded context) and the true location at each timepoint and averaged this distance across all timepoints. The same approach was used to determine context and spatial errors in each environment (familiar or novel). Chance levels were computed by decoding shuffled data and represented on the corresponding figures as a gray dashed line.

#### Decoding using spatial versus mean rate templates

To determine whether context-specific spatial maps carried contextual information, we constructed either location-statified and context-stratified decoding templates (Extended Data Fig. 4a−c, left panel) or a simplified context-only template where solely the mean activity rates across the entire linear track (familiar or novel, respectively) were used to construct population vectors for the decoding template (Extended Data Fig. 4a−c, right panel). We then compared decoding performance on the test runs for each of these templates per dataset by building a ratio between the two obtained error values. A ratio lower than 1 would indicate that the decoding performance of the template using spatial and contextual information was superior to one that used context-dependent firing rate differences only (Extended Data Fig. 4f).

#### Cumulative context decoding performance

Contextual decoding was often incorrect in individual 100-ms time bins, although, on average, it correctly predicted in which environment the animal actually was. We, hence, reasoned that averaging over an increasingly larger number of 100-ms bins should increase the likelihood of decoding the correct context. To get a robust estimate for the timecourse over which such improvement might happen, we randomly drew 75 cells from each recording to perform context decoding as outlined above. We then calculated a decision value for the current context for increasingly longer time intervals (Δ*t*). For each Δ*t*, we divided the test data into non-overlapping segments of length Δ*t* and calculated the average decision value for the current context across all 100-ms bins within this segment. If the mean context decision felt closer to the correct context, that value for that individual segment was 0; otherwise, it was 1. We then averaged over all segments of length Δ*t* to obtain the mean accuracy of context decoding for segments of this size. This procedure was repeated for 15 different random ensembles in each individual recording. The time to 90% decoding accuracy using a fixed sample size of 75 randomly drawn cells was then determined as the first Δ*t* for which the mean context decoding accuracy exceeded 90% (Fig. 3h).

#### Contextual and spatial decoding efficiency

To compare the decoding efficiency between structures and over time relative to their activity, we randomly selected 75 axons/cells and calculated the contextual and spatial decoding efficiency indices using the following formula: log ((actual decoding – shuffled decoding) / transient rate). This procedure was repeated for 15 different random ensembles in each individual recording before being averaged (Fig. 3j,k).

#### Statistics and reproducibility

The reported *n* values indicate numbers of cells and exclude missing (‘NaN’) values. Unless otherwise stated, error bars are showing standard errors of the mean. On whisker plots, boxes are showing 25th to 75th percentiles, and whiskers indicate 99% range. All statistical tests are described in the corresponding figure legends. Unless otherwise stated, statistical comparisons were made either between averages of all cells per dataset or for all cells fulfilling the specific criteria as indicated in the figure legends. No statistical method was used to predetermine sample size. No data were excluded from the analyses. The experiments were not randomized. The investigators were not blinded to allocation during experiments and outcome assessment. To allow the use of ANOVAs on data that initially do not fulfill all the required assumptions (for example, normally distributed population and/or common variance), we used ARTool^80,81^ (available at https://depts.washington.edu/acelab/proj/art/). In brief, this approach offers to apply an additional align-and-rank procedure to the raw data before proceeding with regular ANOVA. This preliminary step ensures that the resulting ANOVA will have main effects and interactions with appropriate type I error rates and suitable power. Furthermore, an additional procedure referred to as ART-C^81^ allows for post hoc pairwise comparisons. In the present paper, similar to previously^26^, we used this tool to analyze correlation values (for example, Fig. 2m−o). When this approach was not necessary, we used two-way or three-way repeated-measures ANOVAs (for example, Fig. 2a−i). In all cases, post hoc pairwise comparisons were performed using Tukey’s test. All comparisons were two-sided, and the null hypothesis was rejected at the *P* < 0.05 level. All statistical analyses, including exact *P* values, are available in Supplementary Table 1.

## Materials availability

This study did not generate new unique reagents.

### Reporting summary

Further information on research design is available in the Nature Portfolio Reporting Summary linked to this article.

## Online content

Any methods, additional references, Nature Portfolio reporting summaries, source data, extended data, supplementary information, acknowledgements, peer review information; details of author contributions and competing interests; and statements of data and code availability are available at 10.1038/s41593-026-02240-0.

## Supplementary information

Supplementary information Supplementary Figs. 1−5

Reporting Summary

Supplementary Video 1 *In vivo* two-photon calcium imaging of LEC axons in the outer molecular layer of the DG while the mouse is navigating through a virtual environment.

Supplementary Video 2 *In vivo* two-photon calcium imaging of MEC axons in the middle molecular layer of the DG while the mouse is navigating through a virtual environment.

Supplementary Video 3 *In vivo* two-photon calcium imaging of DG GCs while the mouse is navigating through a virtual environment.

Supplementary Table 1 All statistical analyses

## Data Availability

Due to the very large size of the original data reported in this paper, the original data will be shared by the lead contacts upon reasonable request.

## Code availability

Custom code used for analyses has been deposited at Zenodo and is publicly available as of the date of publication (10.5281/zenodo.15722268). Any additional information required to reanalyze the data reported in this paper is available from the lead contacts upon reasonable request.
