# Supplementary material for: The dentate gyrus efficiently converges LEC and MEC inputs into multimodal, highly specific and reliable environmental representations
Source: Nat Neurosci. 2026 Mar 27;29(5):1166–80. doi: 10.1038/s41593-026-02240-0 (PMC13156041; doi:10.1038/s41593-026-02240-0)
Supplement: Supplementary file 1 — Supplementary Figs. 1−5 [file 41593_2026_2240_MOESM1_ESM.pdf]

nature neuroscience

Supplementary information https://doi.org/10.1038/s41593-026-02240-0

The dentate gyrus efficiently converges LEC and MEC inputs into multimodal, highly specific and reliable environmental representations

In the format provided by the authors and unedited

Supplementary Fig. 1

a

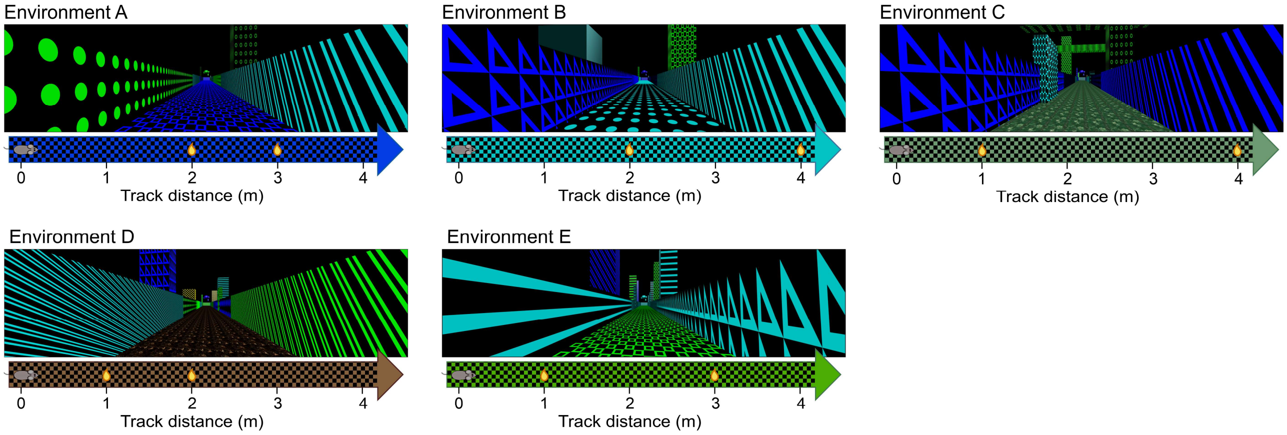

b

Env	Reward 1	Reward 2
A	2	3
B	2	4
C	1	4
D	1	2
E	1	3

c

Pair #	Env 1 (Familiar)	Env 2 (Novel)
1	A	C
2	A	D
3	A	E
4	C	A
5	C	D
6	C	E
7	E	A
8	E	B
9	E	C
10	E	D

Supplementary Fig. 1 | Virtual environments used in Experiment 1.

a In Experiment 1, we used five different virtual environments (A to E) composed of unique combinations of grounds, wall patterns and distant objects. Reward sites were differently distributed in environments, as indicated by the yellow drops on the 4m-long virtual track.

b Table summarizing locations of reward sites depending in each environment.

c Table recapitulating all ten pairs of familiar/novel environments used in Experiment 1. These combinations were pseudo-randomly attributed to the mice.

Supplementary Fig. 2

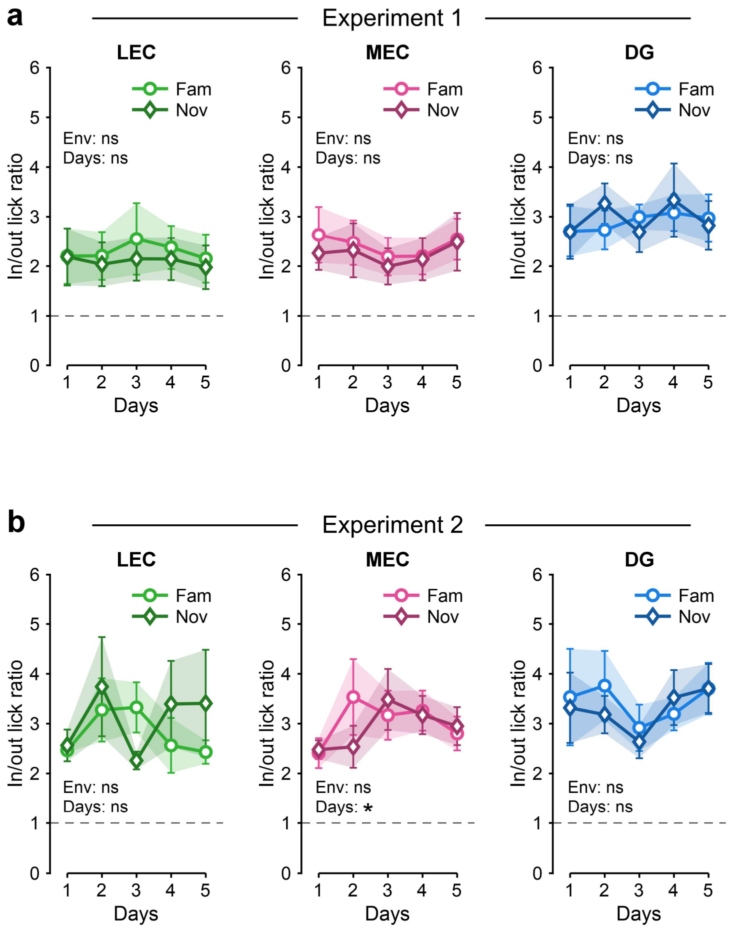

Supplementary Fig. 2 | Licking behavior of mice with labelled LEC-, MEC axons in the DG or GCs in Experiments 1 and 2.

a Ratios between the lick rates observed in the reward zones (regions surrounding the reward sites; **Methods**) and the remaining track. A ratio > 1 indicates that mice are licking preferentially in the reward zones. LEC axons, 6 datasets; MEC axons, 10 datasets; GCs, 7 datasets.

b Similar to **a** for experiment 2. LEC axons, 5 datasets; MEC axons, 7 datasets; GCs, 5 datasets.

a, b Two-way repeated measures ANOVAs, Tukey's post hoc test; ns, not significant; *$P < 0.05$. Lines with shadows indicate mean ± SEM. For exact p values, see **Supplementary Table 1**.

Supplementary Fig. 3

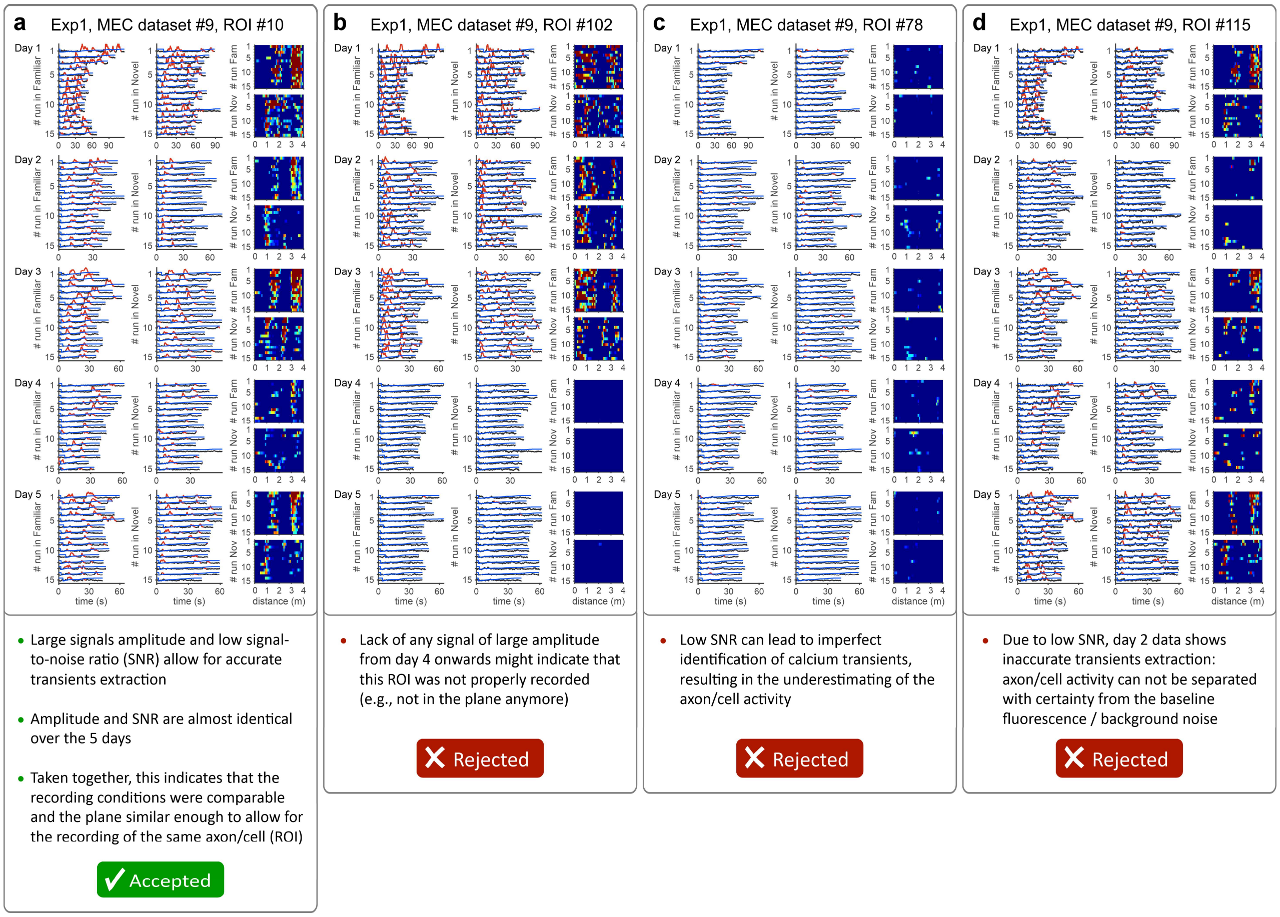

Supplementary Fig. 3 | Description of the manual curation used to select ROIs that were effectively present in the FoV over the entire recording time of 5 days.

Despite the use of state-of-the-art 2P imaging microscopy methods and the help of blood vessels labeling (see **Fig. 1 and Extended Data Fig. 2**), imaging the exact same FoV over multiple days remains challenging, especially for the recording of small structures such as axon terminals. Thus, we manually checked each ROI identified using Suite2P to ensure the sole selection of ROIs of which the activity was reliably tracked over every of the 5 consecutive days of our experiments. Each panel shows the activity of one single ROI from the same (MEC) dataset. Each row of each main panel shows the activity of a single ROI for a different day (from day 1 to day 5, vertically). Left sub-panels, raw calcium traces (grey) with significant transients (red) and linear-track position (blue) over time. Right sub-panels, calcium activity over track distance of the same MEC axon terminal in familiar (top) and novel (bottom) environments.

a Example of a ROI that fulfilled the criteria required for acceptance (see description below the panel) and, thus, was considered for further analyses.

b-d Examples of ROIs that have been rejected and were, thus, not considered for any analysis in this study. See description below each panel for the specific reason leading to rejection of this specific ROI.

Supplementary Fig. 4

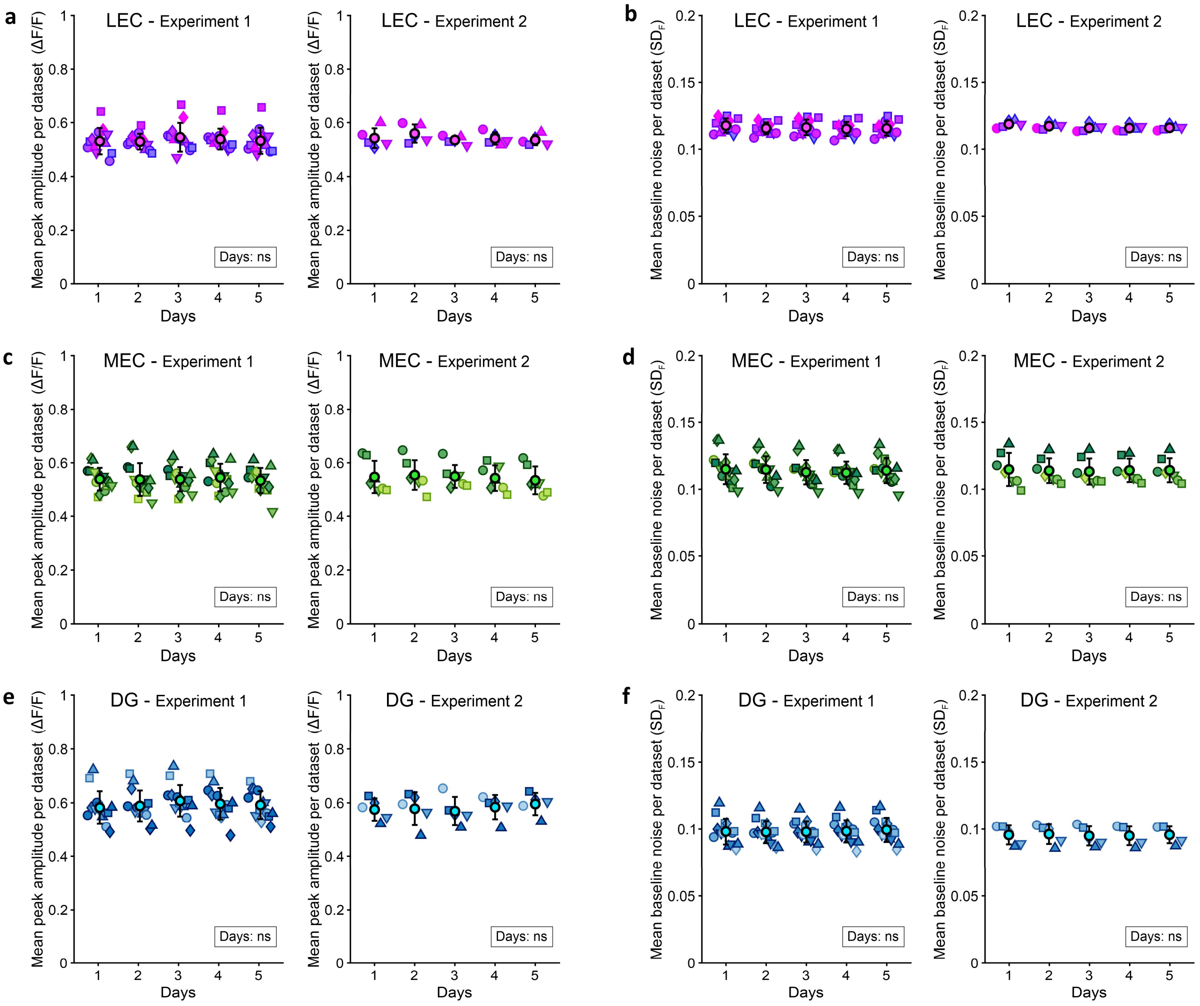

Supplementary Fig. 4 | Peak amplitude and baseline noise were stable over 5 days of imaging for MEC- and LEC axons in the DG as well as GCs in both experiments.

a, c, e Mean peak amplitude of significant transients of individual MEC (**a**) and LEC (**c**) axon terminals and GCs (**e**), averaged per dataset. Left panels, experiment 1; right panels, experiment 2.

b, d, f Similar to **a, c** and **e**, respectively, for baseline noise. Baseline noise is defined as the coefficient of variation of the baseline fluorescence, i.e. the fluorescence signal without significant calcium transients.

a-f One-way repeated measures ANOVAs for days, Tukey's post-hoc test. Each symbol (square, triangles etc.) corresponds to a different animal; circular data points with error bars indicate mean ± SEM. ns, not significant. Datasets: Exp1: LEC, n = 12; MEC, n = 15; DG, n = 14; Exp2: LEC, n = 5; MEC, n = 7; DG, n = 5. For exact p values, see **Supplementary Table 1**.

Supplementary Fig. 5

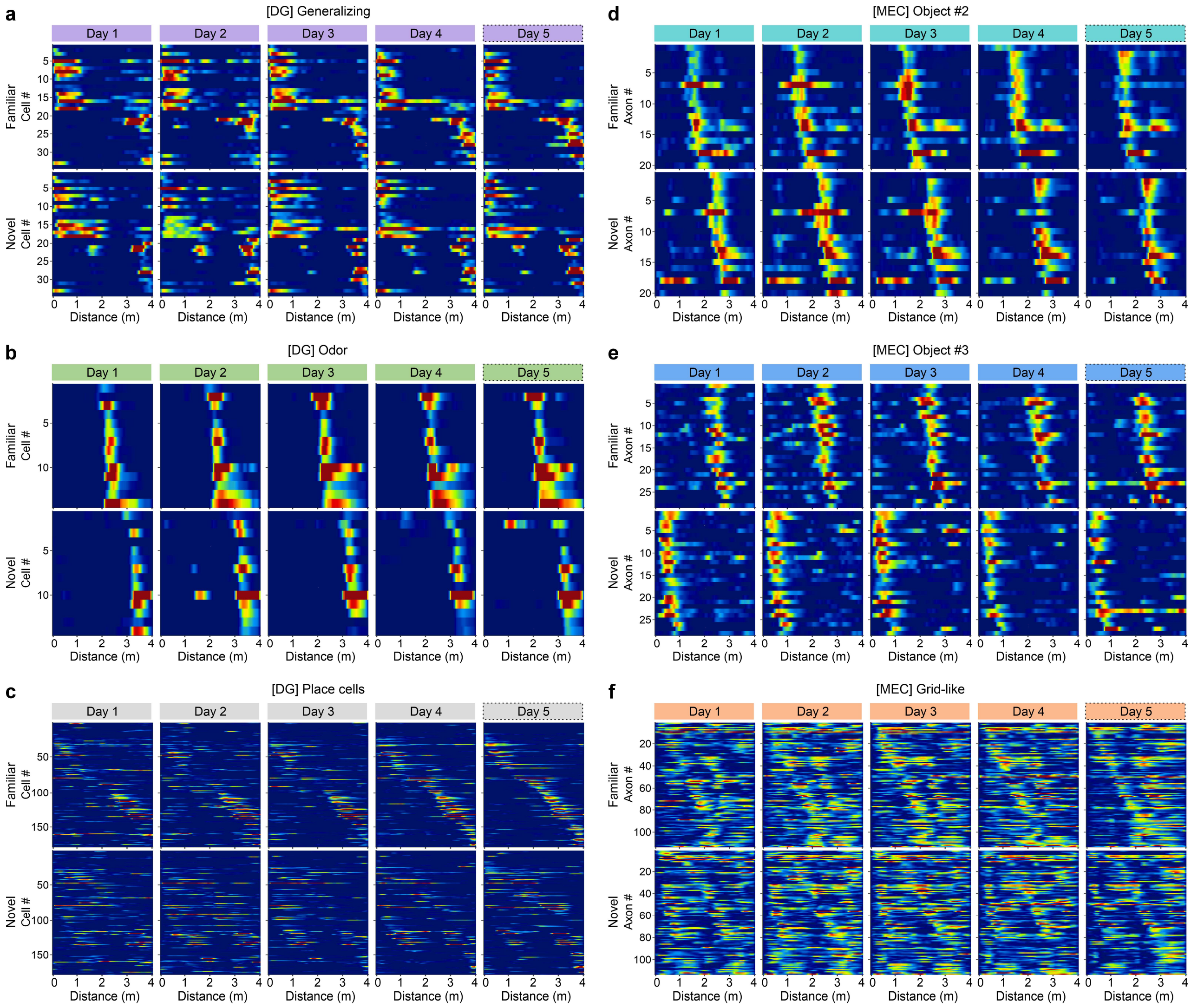

Supplementary Fig. 5 | Activity maps of classes of LEC/MEC axons and GCs (Experiment 2).

a Activity maps of GCs generalizing between the two versions of the environment (i.e. having place field(s) in the same location in both versions of the task), imaged over 5 consecutive days. Sorting according to the cells activity on day 5 in the familiar environment.

b, c Similar to **a** for GCs encoding for the odor (**b**). They have been identified as place cells with a place field(s) in at least one environment but not associated with objects or sensory stimuli (**c**).

d, e, f Similar to **a** for MEC axon terminals with activity patterns specifically associated with object #2 (**d**), or object #3 (**e**) or show grid-like activity patterns (**f**).
