# Supplementary material for: The dentate gyrus efficiently converges LEC and MEC inputs into multimodal, highly specific and reliable environmental representations
Source: Nat Neurosci. 2026 Mar 27;29(5):1166–80. doi: 10.1038/s41593-026-02240-0 (PMC13156041; doi:10.1038/s41593-026-02240-0)
Supplement: Supplementary file 2 — Reporting Summary [file 41593_2026_2240_MOESM2_ESM.pdf]

nature portfolio

Corresponding author(s): Thibault Cholvin, Marlene Bartos

Last updated by author(s): Jan 2, 2026

Reporting Summary

Nature Portfolio wishes to improve the reproducibility of the work that we publish. This form provides structure for consistency and transparency in reporting. For further information on Nature Portfolio policies, see our Editorial Policies and the Editorial Policy Checklist.

Statistics

For all statistical analyses, confirm that the following items are present in the figure legend, table legend, main text, or Methods section.

n/a	Confirmed	
☐	☒	The exact sample size (*n*) for each experimental group/condition, given as a discrete number and unit of measurement
☐	☒	A statement on whether measurements were taken from distinct samples or whether the same sample was measured repeatedly
☐	☒	The statistical test(s) used AND whether they are one- or two-sided<br>*Only common tests should be described solely by name; describe more complex techniques in the Methods section.*
☐	☒	A description of all covariates tested
☐	☒	A description of any assumptions or corrections, such as tests of normality and adjustment for multiple comparisons
☐	☒	A full description of the statistical parameters including central tendency (e.g. means) or other basic estimates (e.g. regression coefficient) AND variation (e.g. standard deviation) or associated estimates of uncertainty (e.g. confidence intervals)
☐	☒	For null hypothesis testing, the test statistic (e.g. *F*, *t*, *r*) with confidence intervals, effect sizes, degrees of freedom and *P* value noted<br>*Give P values as exact values whenever suitable.*
☒	☐	For Bayesian analysis, information on the choice of priors and Markov chain Monte Carlo settings
☒	☐	For hierarchical and complex designs, identification of the appropriate level for tests and full reporting of outcomes
☐	☒	Estimates of effect sizes (e.g. Cohen's *d*, Pearson's *r*), indicating how they were calculated

Our web collection on statistics for biologists contains articles on many of the points above.

Software and code

Policy information about availability of computer code

Data collection	2P imaging data were collected using Scanbox software (Neurolabware)
Data analysis	Data were processed using Suite2p (version 0.14.4; www.github.com/Mouseland/suite2p) and analyzed using MATLAB 2024b (The MathWorks, Inc., Natick, Massachusetts, United States). Custom code used for analyses has been deposited at Zenodo.org and is publicly available at https://doi.org/10.5281/zenodo.15722268.

For manuscripts utilizing custom algorithms or software that are central to the research but not yet described in published literature, software must be made available to editors and reviewers. We strongly encourage code deposition in a community repository (e.g. GitHub). See the Nature Portfolio guidelines for submitting code & software for further information.

Data

Policy information about availability of data

All manuscripts must include a data availability statement. This statement should provide the following information, where applicable:

- Accession codes, unique identifiers, or web links for publicly available datasets
- A description of any restrictions on data availability
- For clinical datasets or third party data, please ensure that the statement adheres to our policy

Original data reported in this paper will be shared by the lead contacts upon reasonable request. All original code has been deposited at Zenodo.org and is publicly

available as of the date of publication (DOI: https://doi.org/10.5281/zenodo.15722268). Any additional information required to reanalyze the data reported in this paper is available from the lead contacts upon reasonable request.

Research involving human participants, their data, or biological material

Policy information about studies with human participants or human data. See also policy information about sex, gender (identity/presentation), and sexual orientation and race, ethnicity and racism.

Reporting on sex and gender	n/a
Reporting on race, ethnicity, or other socially relevant groupings	n/a
Population characteristics	n/a
Recruitment	n/a
Ethics oversight	n/a

Note that full information on the approval of the study protocol must also be provided in the manuscript.

Field-specific reporting

Please select the one below that is the best fit for your research. If you are not sure, read the appropriate sections before making your selection.

☒ Life sciences ☐ Behavioural & social sciences ☐ Ecological, evolutionary & environmental sciences

For a reference copy of the document with all sections, see nature.com/documents/nr-reporting-summary-flat.pdf

Life sciences study design

All studies must disclose on these points even when the disclosure is negative.

Sample size	Data from a total of 40 mice were used. In experiment 1, most animals were recorded twice using two different novel environments to obtain two independent datasets per animal (for a total of 41 datasets; 12 LEC, 15 MEC, 14 DG). In experiment 2, 5 animals were recorded in the LEC, 7 in the MEC and 5 in the DG. Grand totals of LEC axon terminals imaged were of 1310, 1389 MEC axon terminals and 2027 GCs in experiment 1 (mean per session ± SEM: 109.2 ± 9.8 LEC axons; 92.6 ± 6.5 MEC axons; 144.8 ± 11.6 GCs) and in Experiment 2: 431 LEC axons, 632 MEC axons and 650 GCs (mean per session ± SEM:, 86.2 ± 8.3 LEC axons; 90.3 ± 8.4 MEC axons; 130.0 ± 14.4 GCs). Considering the large yield of neuron observations per animal and the high efforts required for animal training and surgery, we have chosen this sample size which is in the range of other studies using similar methodology (see e.g. refs. 49, 79, 89, 90, 93 of the manuscript).
Data exclusions	Animals were excluded from the study if the implanted transcortical window did not permit the reproducible acquisition of high-quality imaging data, e.g. due to intraoperative bleeding or excessive motion artifacts during the experiment.
Replication	Most animals were recorded twice to obtain two independent datasets per animal (for a total of 41 datasets; 12 LEC, 15 MEC, 14 DG), obtained from 6, 10 and 7 animals, respectively), allowing us to reduce the number of animals used.
Randomization	In experiment 1, the two virtual environments used for each individual mouse were randomly picked from a pool of five different virtual environments (see Extended Data Figure 1).
Blinding	The investigators were not blind to allocation during experiments and outcome assessment. Data analysis was performed automatically using the same algorithms and parameters for all data/animals, ensuring unbiased processing, and thus comparability of all data presented in the manuscript.

Reporting for specific materials, systems and methods

We require information from authors about some types of materials, experimental systems and methods used in many studies. Here, indicate whether each material, system or method listed is relevant to your study. If you are not sure if a list item applies to your research, read the appropriate section before selecting a response.

Materials & experimental systems

n/a	Involved in the study
☒	☐ Antibodies
☒	☐ Eukaryotic cell lines
☒	☐ Palaeontology and archaeology
☐	☒ Animals and other organisms
☒	☐ Clinical data
☒	☐ Dual use research of concern
☒	☐ Plants

Methods

n/a	Involved in the study
☒	☐ ChIP-seq
☒	☐ Flow cytometry
☒	☐ MRI-based neuroimaging

Animals and other research organisms

Policy information about studies involving animals; ARRIVE guidelines recommended for reporting animal research, and Sex and Gender in Research

Laboratory animals	We used a total of 40 C57BL/6J wild-type male mice aged 9-12 weeks at the beginning of the experiments.
Wild animals	Not applicable
Reporting on sex	We used male and female mice indifferently. In experiment 1, we used 13 female and 10 male mice. In experiment 2, we used 10 female and 7 male mice.
Field-collected samples	Not applicable
Ethics oversight	All experiments involving animals were carried out according to national and institutional guidelines and approved by the 'Tierversuchskommission' of the Regierungspräsidium Freiburg (license #G22/088) in accordance with national legislation.

Note that full information on the approval of the study protocol must also be provided in the manuscript.

Plants

Seed stocks	n/a
Novel plant genotypes	n/a
Authentication	n/a
